# ReaxFF Force Field Development and Application for Toluene Adsorption on MnMO*_x_* (M = Cu, Fe, Ni) Catalysts

**DOI:** 10.1021/acs.jpca.1c06939

**Published:** 2021-12-09

**Authors:** Vjeran Gomzi, Iva Movre Šapić, Andrej Vidak

**Affiliations:** † Department of Applied Physics, Faculty of Electrical Engineering and Computing, University of Zagreb, Unska 3, 10 000 Zagreb, Croatia; ‡ Department of Physics, Faculty of Chemical Engineering and Technology, University of Zagreb, Marulićev trg 19, 10 000 Zagreb, Croatia

## Abstract

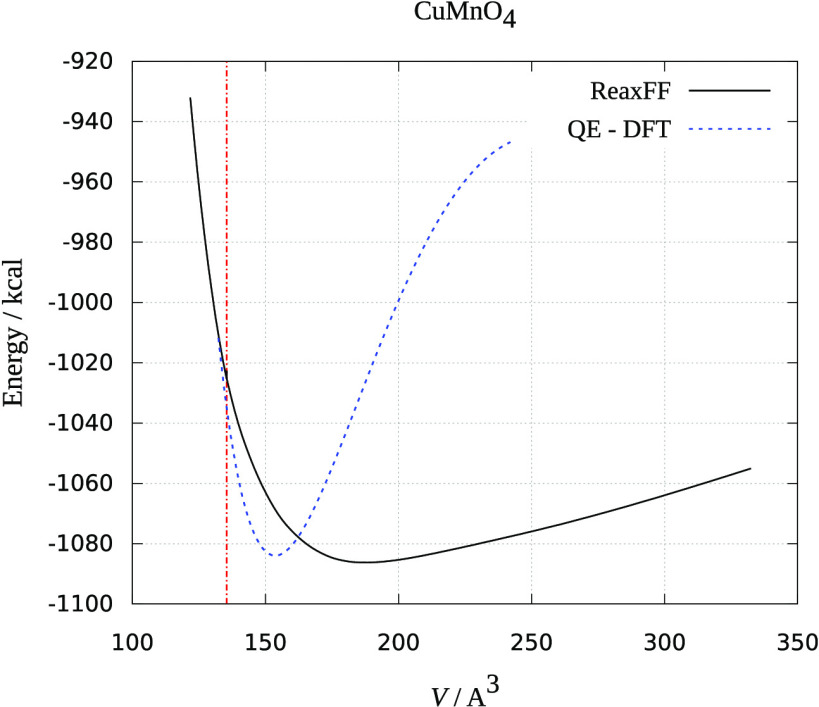

In numerous studies, the application of the molecular dynamics scheme based on the reactive force field (ReaxFF) method has been proven effective in modeling the catalytic behavior of metal–organic compounds. Recently, this method has been successfully applied for M*_x_*O*_y_* (M = Cu, Fe, Mn, Ni) transition-metal oxides. Yet, bimetallic metal oxides of the type MnMO*_x_* (M = Cu, Fe, Ni) were also present in the experimental system but could not be modeled since not all of the force field parameters were available at the time. To bridge this gap, the force field for modeling bimetallic metal oxides had to be developed. Here, we establish the needed force field parameter sets (namely, Cu/Mn/O, Fe/Mn/O, and Ni/Mn/O) and apply them to the problem of toluene adsorption on bimetallic oxide catalyst surfaces to verify their validity. Each training set consisted of at least 10 crystal structures containing at least Cu–Mn–O, Fe–Mn–O, or Ni–Mn–O atoms in contact obtained from the available structure databases. The parameter training has been done using the in-home-compiled version of the ReaxFF code. After training the force fields for geometry reproduction, the parameters were refined using the optimization by atom charges, comparing the ReaxFF values to those obtained for the respective structures using periodic crystal density functional theory (DFT) codes. The as-developed force fields were then applied to the process of toluene adsorption/degradation on MnMO*_x_* catalysts. Results obtained show agreement with previous experimental expectations, although some remarks are given since the initially presumed crystal structure of bimetallic oxide Mn_1–*x*_M*_x_*O*_y_* crystallites may still have an impact on theoretical predictions. The presented are, to the best of the authors’ knowledge, the first applications of the ReaxFF approach to the Mn–(Cu|Fe|Ni)–O–C–H interaction.

## Introduction

To design the process for removing volatile organic compounds (VOCs) from the environment, we study reactions to degrade complex organic compounds present in wastewater to CO_2_ and H_2_O. To take account of the economy and feasibility of the complete technological cycle, the use of catalysts to speed up and/or enable reactions is appropriate. The step further in the production-degradation economy is to take into account the availability and the price of the catalyst production process. The balance between catalytic performance and production costs motivates us to use less expensive and readily available catalysts with catalytic activity comparable to those of the commonly used more expensive ones. Thus, the motivation is to replace noble-metal-based catalysts (such as Pt or Au) with much more available transition-metal oxide catalysts (such as these based on Cu, Fe, Ni, and Mn) as the latter are shown to have similar activity for reactions of interest. This basic approach is explained further below with specific reference to the problem at hand.

Removal (i.e., adsorption and degradation) of VOCs from the environment is greatly enhanced by the use of catalysts. Optimal catalysts are at the same time efficient, easy to obtain, and affordable to produce. Manganese oxide catalysts and especially MnMO*_x_* (M = Cu, Fe, Ni) bimetallic transition-metal oxides were proven to be prospective catalyst candidates for processes carried out at relatively low temperatures and of activities comparable to much more costly alternatives.^1−3^ In our recent work, we used theoretical modeling to support and broaden the understanding of such catalytic process taking place.^1^ Regarding the choice of the theoretical approach for such research, the Reactive Force Field (ReaxFF) method is a molecular dynamics (MD) approach fit specifically to study reactions.^4^ Furthermore, the method has been well proven in modeling the catalytic degradation on metal catalytic centers.^5^ The method has also been shown to perform well in the mentioned recent research of catalytic activity of several MnMO*_x_* (M = Cu, Fe, Ni) catalysts within our group.^1^ In course of that work, we could model only single metal oxides (such as CuO, Fe_2_O_3_, MnO_2_, and NiO) since the parameters involving the interaction of atoms of different transition metals in question (i.e., Mn with each of Cu, Fe, Ni) were at the time nonexistent, although the presence of both bimetallic and single transition-metal oxide phases was experimentally found for all MnMO*_x_* (M = Cu, Fe, Ni) samples. Thus, our previous investigation raised interest in developing parameters for the calculation of bimetallic oxide species, which is the subject of this work.

Being the molecular dynamics-based method, the applicability of ReaxFF calculations is limited by the existence of parameters among interacting atoms.^4,5^ In contrast to other MD methods, parameters are unique for the atom pairs and not dependent on the type of bond between these in molecule or structure.^6^ Here, we perform optimization of metal–metal parameters in the bimetallic oxide crystal environments but taking into account that our goal is to model specifically interactions of such combined crystals with the organic molecules (toluene).

Special interest in toluene degradation catalysis lies in the fact that toluene (methylbenzene) is a well-known pollutant present in many common products. It is a harmful compound that is an ingredient of everyday chemicals such as petrol, lacquers, and dyes.^7^ According to NPI,^8^ among others, benzaldehyde and cresol are toluene degradation products in air, which are also harmful to humans. It is thus of interest to regulate toluene removal even before it evaporates from water.

## Methods

The outline of methods to produce, validate, and use the three force fields is as follows. First, for a set of training data, MD calculations (i.e., ReaxFF calculations) were done at a temperature of 1 K. In successive training, calculation series’ temperature is set to 300 K and eventually 500 K. This is done in training of each of the three force fields to be developed (Cu/Mn/O, Fe/Mn/O, and Ni/Mn/O). Then, calculations were done for specific crystals of interest (those that we hope to compare to experimental data, albeit industrially crude): MnFeO_3_, NiMnO_3_, and CuMnO_4_. These calculations were followed by the crystal volume variations, which were compared to the density functional theory (DFT) energy calculations for the validation of force fields. Finally, we calculated crystallites with several toluene molecules and compare the results to experimental data.^1^

Following the established protocol to train the parameters involved in the ReaxFF potential functions, these have to be parametrized against experimental results and high-level ab initio calculations.^9^ For a complete description of the ReaxFF field, all of the factors in expansion should be optimized during the force-field optimization process.^10^ The ReaxFF potential is written as
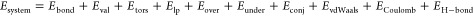
where *E*_system_ is the overall interaction energy of the system; *E*_bond_ is the bond energy, the two-body attractive term that is dynamically dependent on bond orders of specific bond type, σ, π, or ππ; *E*_val_ and *E*_tor_ are the three-body valence angle terms and the four-body torsion terms, respectively, describing angle and torsion strain energy; *E*_lp_ and *E*_over/under_ are the energy contributions from lone-pair electrons, and the penalty energy coming from overcoordination and undercoordination; *E*_conj_ represents the conjugation energy term; *E*_vdWaals_ is the energy of van der Waals interactions; *E*_Coulomb_ is the electrostatic contribution; *E*_H-bond_ represents hydrogen bond; and *E*_Coulomb_ and *E*_vdWaals_ are the nonbonded interactions between all pairs irrespective of the connectivity in the system. The Coulomb charge is calculated dynamically from the electronegativity equalization method.^4,6,10−12^

For Cu/Mn/O and Ni/Mn/O parameters, we choose at least 10 crystal structures from Crystallographic Open Database^13^ consisting of at least Cu, Mn, and O or Ni, Mn, and O atoms, respectively (see Table 1). For Fe/Mn/O parameter training, a training set of 39 structures consisting of at least Fe, Mn, and O atoms, obtained in their crystal form from the Materials Project database,^14^ was used (Table 1). Each step in force-field parameter optimization has been done performing MD energy minimization of the calculated structure consisting of 5000 steps of 0.1 fs using the NVT/Berendsen thermostat with a damping constant of 100 fs. The optimization of the parameters has been done using the parabolic search as implemented in the original van Duin code^4^ with small in-home modifications to the code. First, force-field optimization is performed at temperature set at 1 K. Initial parameters are then reoptimized using the same training set and other parameters at temperatures 300 and 500 K. Force-field optimizations were first done comparing crystallographic and ReaxFF geometries and then comparing atom charges as obtained from DFT and ReaxFF calculations (see description below).

**Table 1:** List of Geometry and Charge Data Points for Fitting Parameters in Each Training Set

parameter set	#structures in geometry training set	#geometry data points	#structures in charges training set	#charge data points	#fitted parameters in force field^a^
Cu/Mn/O	10	1863	10	640	20
Fe/Mn/O	39	3117	13	152	20
Ni/Mn/O	11	3498	11	1161	20

a See text for explanation.

Force-field optimizations performed involved evaluating the error of structural differences between the ReaxFF-obtained result and the original crystallographic structure. The mean total number of geometry data points for each parameter set exceeded greatly the number of parameters to be fitted (see Table 1). For metal–organic structures that were used in training (see the Supporting Information, SI, for complete structure lists), we chose to include a complete structural description for developing the force fields because we wanted the fitted parameters to perform best in exactly such environments. Parameters are optimized in a sequential manner, as implemented in the original van Duin code.^4,15^ During optimization, the error of ReaxFF MD geometries was at all times evaluated by comparison to the initial (crystal) geometries, rather than evaluating error between subsequent ReaxFF MD geometries. This was chosen so in an attempt to train the parameters in optimization to fit experimental data as much as possible.

Initial parameters for ReaxFF potentials for Fe were taken from ref (16), for Cu/N from ref (17), for Ni from ref (10), and for Mn/O/C/H from ref (18). All of the initial force fields have retained their validity for modeling the reactions of transition-metal oxides with organic molecules since the M/C/O/H (M = Cu, Fe, Mn, Ni), parameters were the same as in the previously developed force fields chosen here for enlarging (see refs (10, 16−18)). All of the general parameters in the initial and final force fields, as well as the proposed application of the developed force fields suggest that these would be classified as belonging to the combustion branch.^5^

The initial force field parameters were determined by combining the original metal-hydrocarbon parameter sets (Mn–C–O, Ni–C–O, Fe–C–O, Cu–C–O) with the trial parameters for new bonds (Cu–Mn, Fe–Mn, and Ni–Mn) and respective off-diagonal elements. Final parameters are obtained by training of these three parameter sets to crystallographic and QM data for chosen systems relevant for the description of the MnMO*_x_* (M = Cu, Fe, Ni) catalyst surface in the optimization process. The optimization of the parameters was carried out via a single-parameter search optimization to minimize the following sum of squares^15^
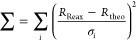
where *R*_theo_ is the literature value, *R*_Reax_ is the ReaxFF calculated value of the corresponding value, and σ is the constraint weight specified in the training set.^19^ Thus, optimal parameters (minimum ∑) result from the best reproduction of the *Z*-matrix representation of training structures obtained in the successive ReaxFF MD runs.

For chosen structures, partial charges were obtained from density functional theory (DFT) calculations on periodic structures using Abinit^20^ and Quantum Espresso^21^ codes. Calculations were performed using self-consistent field (SCF) calculations using the projector augmented wave (PAW) pseudopotentials on crystal structures from crystallographic data. Atomic charges used in training were integrated Hirshfeld charges^22^ calculated from the obtained wavefunctions by the use of *Cut*3*D* and *Critic*2^23^ postprocessing utilities for *Abinit* and *Quantum Espresso*, respectively. A list of such obtained charges is given in the SI. After the initial training of the force fields for geometry reproduction, the ReaxFF parameters were refined using the force-field optimization by atom charges, comparing the ReaxFF values to those obtained for the respective structures using periodic crystal DFT codes. The number of such charge data points for a specific set of atomic parameters is stated in Table 1.

(Somewhat modest training set of structures is the direct result of the number of available structures in named databases and the available ReaxFF force fields: other, usually more complex structures were found by database search, but these were consisting of elements not having developed parameters for M (M = Cu, Fe, Ni) and Mn available.)

Parameters included in the optimization (last column in Table 1 above) were in all cases (refer to the SI, and refs (11) and (24) for a detailed description of the Reax force field parameters)

- 14 bond energy parameters (*Edis1*; *Edis2*; *Edis3*; *LPpen*; *pbe1*; *pbo5*; 13*corr*; *pbo6*; *pbe2*; *pbo3*; *pbo4*; *pbo1*; *pbo2*; *ovcorr*, as marked in the standard form of ReaxFF force field).
- 6 off-diagonal terms (*Ediss*; *Ro*; *γ*; *rsigma*; *rpi*; *rpi2*, as marked in the standard form of ReaxFF force field).

Additional angle and torsion terms were not fitted since they are not considered for the calculation of metal–metal bond energies within the ReaxFF MD method.^25^ Initial parameters describing Mn–Cu and Mn–Ni interactions were taken from Mn–Fe parameters, which were first obtained on the largest geometry training set. Initial parameters for Mn–Fe bonds were initially taken to be the same as Mn–Mn parameters and put into the force-field optimization process.

**Scheme 1:**
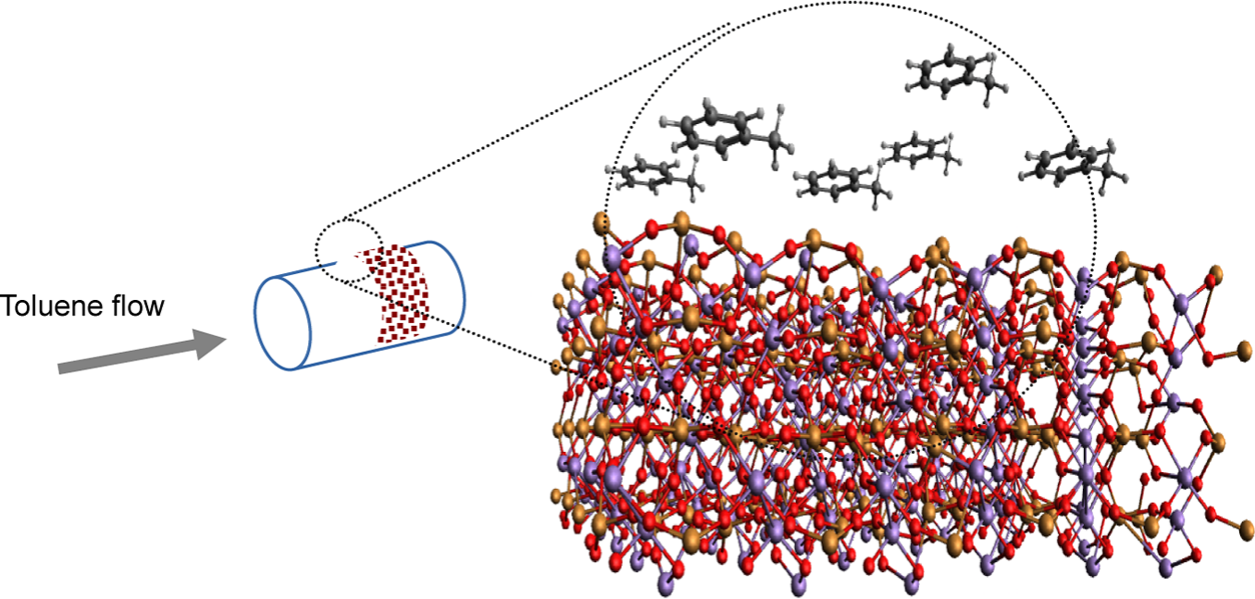
Model System

It is important to note, however, that the particular model (see also Scheme 1) of force-field application here is to establish as well as make possible the expected structure of the bimetallic manganese–metal oxides, as well as the charge distribution on the bimetallic oxide slab surface. Reactions that take place are mostly expected to appear among carbon and hydrogen atoms of the toluene with the slab. Thus, the applied force field is mostly targeted to describe crystal metal oxide structure in interaction with the surrounding hydrocarbon molecules. Yet, to our benefit, all of the hydrocarbon–individual metal-oxide interactions were already described by respective force fields used for our enlarging here.^10,16−18^ Such a method of taking into account the exact process to be modeled while reoptimizing the ReaxFF force field has also been applied elsewhere.^26^ This is, as will be seen shortly, only an approximation, since the slab surface molecules are shown to participate more or less largely in reactions, especially for higher modeling temperatures.

To initially validate the obtained force fields, we performed ReaxFF MD calculations of CuMnO_4,_ FeMnO_3_, and NiMnO_3_ crystals while slightly changing the unit cell parameters. For chosen points in these MD energy minimizations, DFT (Quantum Espresso) calculations were performed to get respective self-consistent energies. The size of the crystal unit cell has been modified by simultaneous increase or decrease of all of the unit cell lengths (*a*, *b*, and *c*) by 0.0004 Å in successive MD steps using *vregime.in* volume modification ReaxFF capability. Cell size changes were initiated only after 15 000 steps of 0.25 fs MD simulation using NVT/Berendsen thermostat with a damping constant of 100 fs at 1 K of a large 7 × 7 × 7 crystal supercell obtained from crystallographic databases. From these calculations, energy minimum is located and six structures (of each crystal species) are identified. For these, Quantum Espresso inputs are prepared and SCF DFT calculations are performed on these structures. We used the latest projector augmented wave (PAW) pseudopotentials for Cu, Fe, Mn, Ni, and O as available from the *Quantum Espresso* pseudopotentials database, all of which were generated using “atomic” code by A. Dal Corso v.6.3.^27,28^ During the calculation, Marzari–Vanderbilt–DeVita–Payne cold smearing has been applied.^29^

### Application to Toluene Degradation

Following initial motivation, the ReaxFF parameters obtained by force-field optimization were used for modeling toluene adsorption and degradation on bimetallic catalyst surfaces. A quite simplified model system for the experimental setup is shown in Scheme 1. Gaseous toluene is flowing through a tube in which a powder catalyst is placed. A model consists of a metal-oxide crystallite particle with a vacuum layer to which toluene molecules are added. The greatest simplification is of course in the size of the model and the presence of only one metal-oxide species at a time, whereas, as found by X-ray diffraction (XRD), in each of the setups, a variety of metal-oxide crystallites are present.^1^

Upon obtaining and validating force-field parameters, ReaxFF MD simulation for toluene adsorption is performed for FeMnO_3_, CuMnO_4_, and NiMnO_3_, which are found to be the most abundant bimetallic oxide forms by XRD.^1^ The six toluene molecules were positioned in the 20–30 Å vacuum layer added to the respective metal oxide crystallite consisting of three or more unit cell repeats in two dimensions and at least two in the third. Complete structures consisted of approximately 1100–1500 atoms (see also structures in the SI). Calculation was then performed of 100 000 steps of 0.25 fs using an NVT/Berendsen thermostat set at 500 K, modeling in total 25 ns of adsorption/degradation process time.

## Results and Discussion

**Figure 1:**
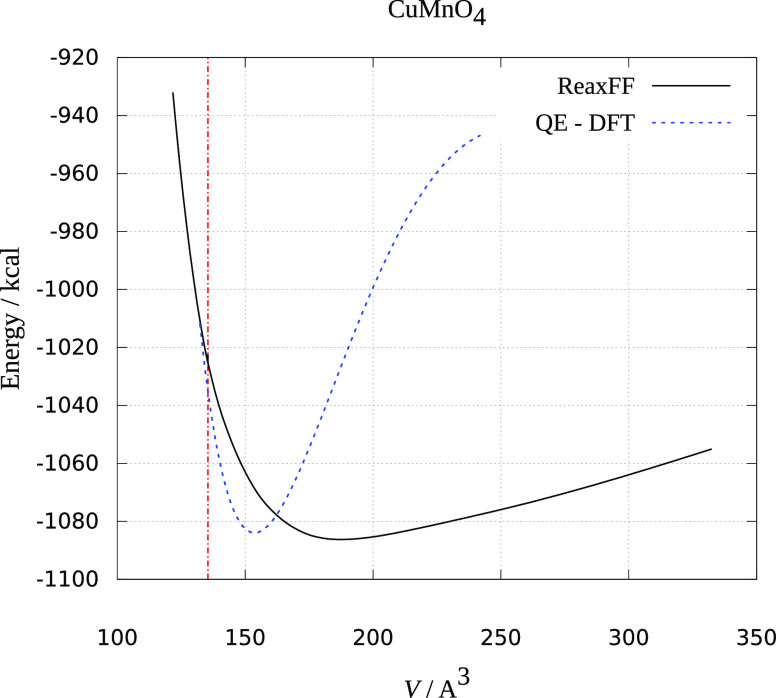
Dependence of ReaxFF and DFT energy on the volume of the CuMnO_4_ unit cell. The red dotted line is the experimental unit cell volume.

**Figure 2:**
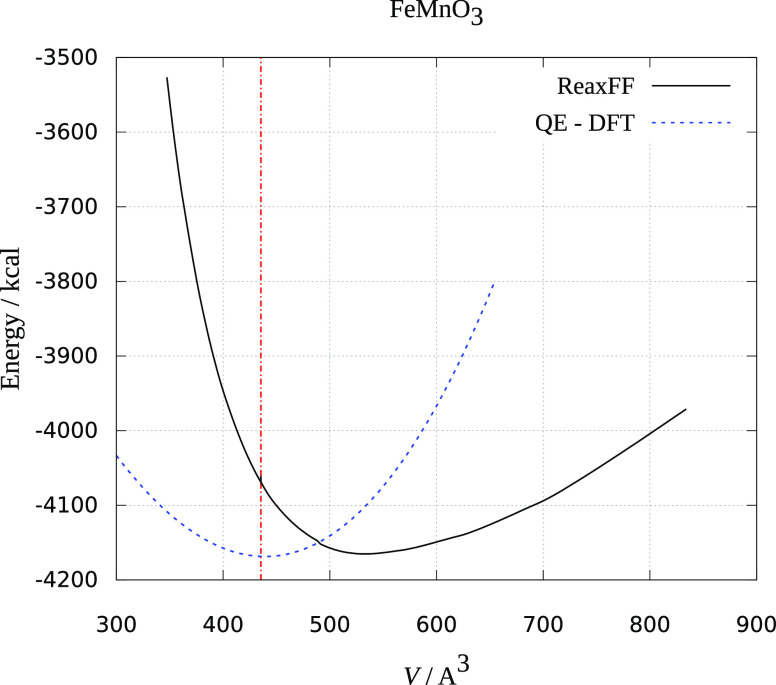
Dependence of ReaxFF and DFT energy on the volume of the FeMnO_3_ unit cell. The red dotted line is the experimental unit cell volume.

**Figure 3:**
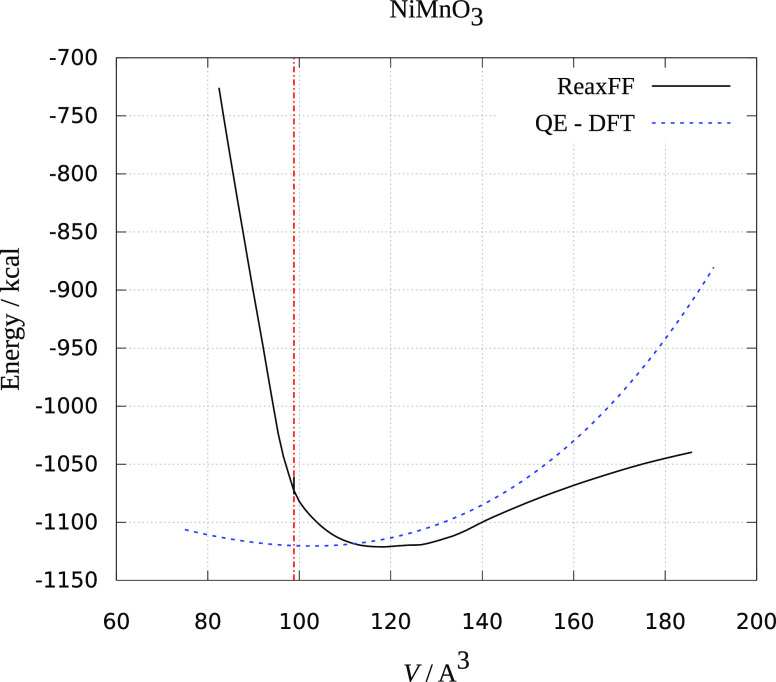
Dependence of ReaxFF and DFT energy on the volume of the NiMnO_3_ unit cell. The red dotted line is the experimental unit cell volume.

The force-field parameter sets for Mn/Cu/O/C/H, Mn/Fe/O/C/H, and Mn/Ni/O/C/H as obtained by the above protocol are available for download as Supporting Information. In an attempt to validate these parameters, the energy dependence on the size of the unit cell for CuMnO_4_, FeMnO_3_, and NiMnO_3_ crystals (equation of state-like diagrams) is obtained from ReaxFF calculations and compared to the periodic DFT code energy calculations. The results are presented in Figures 1–3. Crystal volume from crystallographic data is shown as a vertical red dashed line. It is evident that all of the ReaxFF calculations predict an increase in energy upon volume contraction. Upon unit cell increase, both ReaxFF and DFT methods show minimum close to the crystallographic volume (DFT energies are scaled to be presented in the same graphs and fitted to the Birch–Murnaghan equation of state^30^). As seen from Figures 1–3, the deviations from experimental and theoretical data are not large, and the comparison supports the reliability of the obtained force fields in reproducing MnMO*_x_* (M = Cu, Fe, Ni) the crystal behavior reasonably well.

### Application on Modeling Toluene Degradation and Comparison to Experimental Data

**Figure 4:**
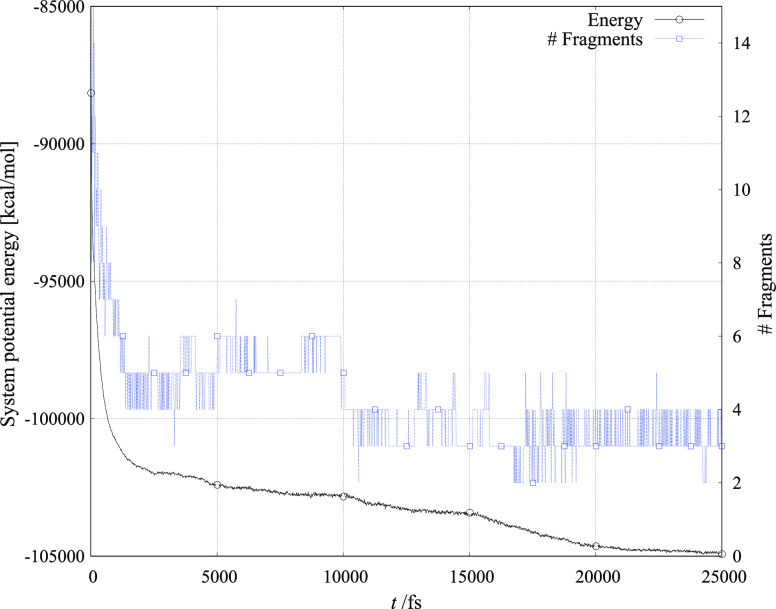
Simulation of CuMnO_4_ crystallite reacting with six toluene molecules.

**Figure 5:**
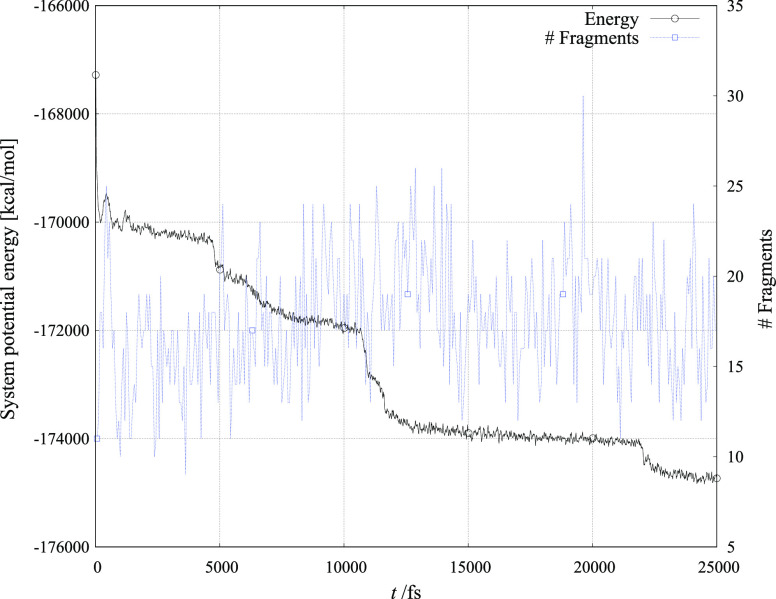
Simulation of FeMnO_3_ crystallite reacting with six toluene molecules.

**Figure 6:**
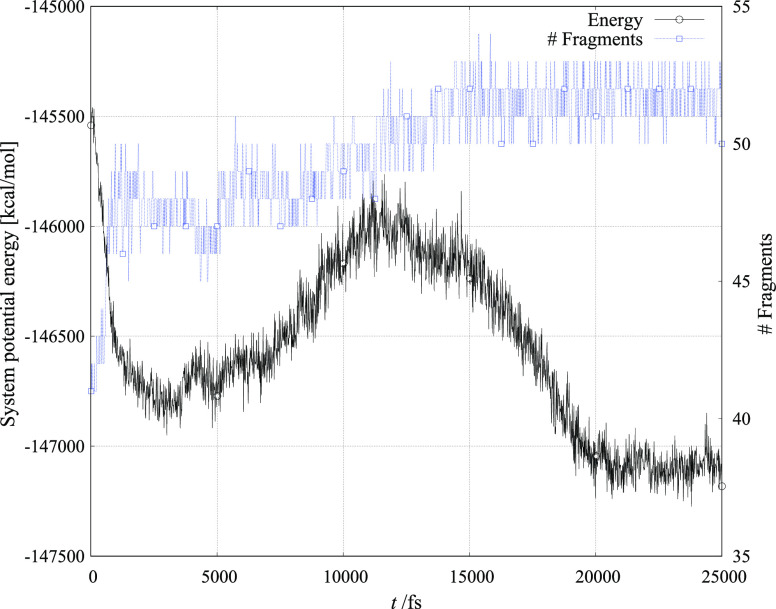
Simulation of NiMnO_3_ crystallite reacting with six toluene molecules.

Figures 4–6 show the evolution of total ReaxFF calculated energy and the number of molecular fragments during the 25 ns simulation period for the three crystallite systems with six toluene molecules added to the vacuum layer.

The number of atoms that constitute a molecular fragment is of course dependent on the distance of the atom cluster from other atoms and molecules in simulation. From the figures, it is observed that both the variation in system energy and the number of fragments are largest in structures that are least predisposed for catalytic activity. Furthermore, the number of fragments decreases most monotonically in the structure that is experimentally and within our simulation found to be the best catalyst candidate.

Inspired by other theoretical investigations of hydrocarbon catalysis on metal catalysts,^31^ we also note the number and distribution of fragments specia for the three model systems at the end of simulation (Table 2).

**Table 2:** Fragment Compositions at the End of MD Simulation for the Three Catalysts

catalyst	structures present (number of molecules)
CuMnO_4_	O_2_ (1)
	C_7_H_8_O (1)
FeMnO_3_	Mn (2)
	H_2_O (1)
	C_7_H_8_ (1)
	MnO (9)
	Mn_2_O (1)
	MnOH (2)
NiMnO_3_	O (15)
	O_2_ (23)
	C_7_H_8_ (5)
	MnO_2_ (1)
	MnO_3_ (2)
	Mn_3_O_7_ (1)
	C_7_H_8_O (1)

It is seen that for efficient activity, the number of fragments is even more important than the number of molecular species (compare data for FeMnO_3_ and NiMnO_3_). Also, a large number of oxygen atoms and molecules indicates a rather fluctuating NiMnO_3_ surface, which under the modeling conditions seems not to be the best environment for toluene adsorption. The fluctuations in this system are also visible in Figure 6, where these are represented by large variations in potential energy throughout the simulation. The fact that this NiMnO_3_ behavior may not be a mere consequence of imperfections of our theoretical approach, but rather the feature of this (highly correlated, magnetic) system itself, may be supported by another recent theoretical investigation.^32^

**Figure 7:**
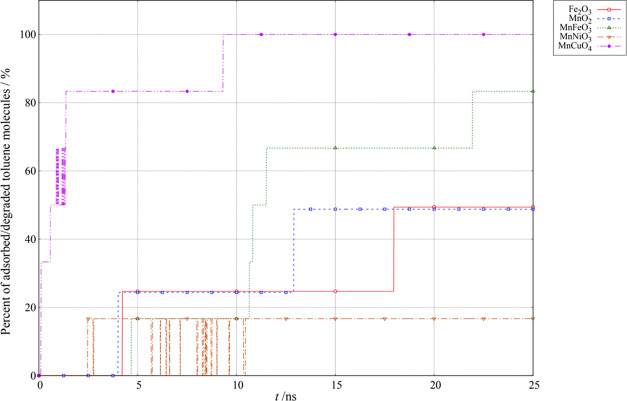
Comparison of predisposition of activity for toluene removal: 25 ns ReaxFF simulation for Fe_2_O_3_, MnO_2_, MnFeO_3_, CuMnO_4_, and NiMnO_3_ at 500 K using the trained parameter sets.

Previously published work has modeled well the actual experimental results obtained from the XRD and X-ray photoelectron spectroscopy (XPS) results.^1^ From these measurements, it has been found that for the investigated catalyst compositions, oxides that contribute to (catalytic, adsorption, and/or degradation) reaction processes the most are expected to actually be single metal oxides, as these are found to be the most abundant in the samples. However, several bimetallic species, although of less abundance, still contribute. Here, we show that bimetallic metal-oxide species have the potential for a positive impact on overall catalyst activities. Their effect is possibly dependent on the bimetallic oxide preparation methods since these affect most the actual catalyst composition. The potential for catalytic activity on MnMO*_x_* (M = Cu, Fe, Ni) oxides as obtained from ReaxFF by the use of the developed force fields is shown in Figure 7. The potential for catalytic activity is estimated by the percentage of adsorbed or oxidized toluene molecules, i.e., by the expression
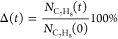
where Δ(*t*) is the value shown on the *y*-axis, *N*_C_7_H_8__(0) is the initial number of added toluene molecules; in our case, this number is equal to 6, and *N*_C_7_H_8__(*t*) is the number of free toluene molecules at time *t* during the simulation. The activity of the most active single metal oxide specia, Fe_2_O_3_ and MnO_2_, calculated in the previous work^1^ are shown for reference.

Our results are in reasonable agreement with the experimental investigations of bimetallic ferrite–manganese oxide catalysts for toluene degradation done by another group.^33^ In our previous report,^1^ considerable activity is, however, in both experimental evaluation and theoretical model expected for single-phase oxides too (see the catalytic activities of MnO_2_ and Fe_2_O_3_ in Figure 7 for reference). The reason for this discrepancy between the two investigations may lie in different sample preparation techniques applied or calcination/postprocessing of the samples and measurement conditions but certainly calls for further investigation. It is however common finding in both of the mentioned investigations that an increased amount of bimetallic oxide specia should enhance activity. The amount of bimetallic oxides species within the sample (as well as the exact catalyst composition) is expected to be modified by initial preparation and subsequent calcination of the compounds. Thus, the well-known activity-stability compromise may also come into play upon designing such bimetallic oxide catalysts.

Even though optimization and application were performed under all of the above approximations, the obtained force fields manage to reproduce the experimental findings remarkably well. The error margin is however rather high since the experiments that we compare to are rather qualitative, due to their setup (structures are loosely defined only by the net composition, not by the spatial distribution of atoms or oxide phases). Thus, the present force fields are shown to be capable of reproducing some of the industry-grade applications. For finer experiments, tuning of presented parameters is certainly to be expected and advised.

## Conclusions

Parameters for the application of the ReaxFF MD method for the theoretical investigation of bimetallic Mn–Cu, Mn–Fe, and Mn–Ni oxides were developed by training them to DFT and crystallographic data, with special emphasis for reproducing structure and charge distribution. As confirmed by initial validation comparing energy–volume diagrams for (CuMnO_4_, FeMnO_3_, and NiMnO_3_), the structures predicted by MD calculation using the obtained parameters agree reasonably well with the theoretical (DFT) structures.

ReaxFF calculations of bimetallic oxides in interaction with toluene using developed parameters show agreement with experimental findings for the catalytic activity of modeled compounds. This opens the possibility for *in silico* search for optimal catalyst structure and/or composition. Some caution is advised since the parameters were not thoroughly tested for the prediction of crystal structure changes when the Mn (or other constituent metal) content changes in Mn_1–*x*_M*_x_*O*_y_* oxides.

The presented are, to the best of the authors’ knowledge, the first applications of the ReaxFF approach for Mn–(Cu|Fe|Ni)–O–C interactions.

## Supporting Information Available

List of structures used, and final force field parameters for three newly developed force fields (SI1) (PDF)
Full list of all of the geometries and DFT charges of crystal structures and clusters used in force-field training (SI2) (TXT)
Initial and final structures of the ReaxFF MD calculation of toluene adsorption on bimetallic oxides using the developed force fields (SI3) (TXT)
Parameter sets in text format (SI4) (TXT)
