# Supplementary material for: ReaxFF Force Field Development and Application for Toluene Adsorption on MnMOx (M = Cu, Fe, Ni) Catalysts
Source: J Phys Chem A. 2021 Dec 9;125(50):10649–56. doi: 10.1021/acs.jpca.1c06939 (PMC8713286; doi:10.1021/acs.jpca.1c06939)
Supplement: Supplementary file 1 — jp1c06939_si_001.pdf [file jp1c06939_si_001.pdf]

Supporting information to:

ReaxFF force field development and application for toluene adsorption on MnMOx (M=Cu, Fe, Ni) catalysts

Vjeran Gomzi[a], Iva Movre Šapić[b], Andrej Vidak[b]

vjeran.gomzi@fer.hr

[a] *Applied physics dept., Faculty of electrical engineering and computing, Unska 3, 10 000 Zagreb, Croatia*
[b] *Physics dept., Faculty of Chemical Engineering and Technology, Marulićev trg 19, 10 000 Zagreb, Croatia*

Training sets:

Cu/Mn/O/C/H

COD Id	Structure
1526929	CuMn2O4
1530385	CuMn2O4
1533677	Cu1.4Mn1.6O4
2106773	Cu2Mn3O8
2227420	C10H14Cu2MnN4O14
4109045	C78 H230Cu17Mn28N12O128
4326303	C14 H26CuMnN2O13
7216595	C3H10Cu0.5Mn0.5O8
9000847	Cu4H14MnO18S2
9011507	Cu1.04Mn0.96O2
9011834	CuMnO2

Fe/Mn/O/C/H

MaterialsProject ID	Structure
mp-1221857	Mn4FeO5
mp-754642	Mn2FeO4
mp-756562	Mn3FeO8
mp-761563	Mn2FeO3
mp-532236	Mn17Fe13O40
mp-753618	Mn9FeO10
mp-757061	Mn4FeO8
mp-776272	Mn5FeO12
mp-1222437	Mn5(FeO3)4
mp-771188	Mn13Fe3O32
mp-758400	Mn5(FeO3)4
mp-761716	MnFe2O3
mp-38856	Mn2FeO4
mp-754962	Mn2FeO6
mp-674482	MnFeO3
mp-18750	Mn(FeO2)2
mp-1176616	Mn3FeO8
mp-773198	Mn5Fe3O16
mp-1221795	MnFe4O5

mp-33708	Mn(FeO2)2
mp-762538	Mn13Fe11O32
mp-1221655	MnFeO4
mp-769622	Mn9(FeO6)4
mp-777317	Mn7Fe3O20
mp-753479	Mn3Fe3O8
mp-756788	Mn3FeO8
mp-1221648	MnFeO2
mp-1221814	MnFe5O8
mp-755276	Mn5FeO12
mp-761315	MnFeO4
mp-771261	Mn3FeO8
mp-759744	MnFeO3
mp-1221773	MnFe2O3
mp-757698	Mn23FeO32
mp-697842	Mn(FeO2)2
mp-757662	Mn3Fe5O12
mp-753787	Mn2FeO6
mp-752751	Mn2FeO6
mp-706492	Mn19Fe17O48

Ni/Mn/O/C/H

COD Id	Structure
1530384	Mn2NiO4
1541565	MnNi0.5O4V1.5
4118052	C33H24Mn0.5Ni1.5O16
5910161	Mn2NiO4
7115434	C68H140Mn24Ni2O84
7203362	C4H6Mn0.79Ni0.21O6
9013975	MnNi6O8
9013976	Mn0.8Ni6.4O8
9013977	Mn0.6Ni6.8O8
9013978	Mn0.4Ni7.2O8
9013979	Mn0.2Ni7.6O8

Cu/Mn/O/C/H Force field

Refinement of:
Cu: Reactive Dynamics Simulation of Monolayer and Multilayer Adsorption of Glycine on Cu(110) Susanna Monti*a, Cui Li b and Vincenzo Carravetta b
Mn: Electrode-electrolyte interface layers in lithium ion batteries using reactive force field based molecular dynamics by Sahithya Reddivari, doctoral thesis, 2016, University of Michigan

Reactive MD-force field: Mn/Cu/O/C/H/N Gomzi et al 2021.

```
 39       ! Number of general parameters
  50.0000 !Overcoordination parameter
   9.5469 !Overcoordination parameter
   1.6725 !Valency angle conjugation parameter
   1.7224 !Triple bond stabilisation parameter
   6.8702 !Triple bond stabilisation parameter
  60.4850 !C2-correction
   1.0588 !Undercoordination parameter
   4.6000 !Triple bond stabilisation parameter
  12.1176 !Undercoordination parameter
  13.3056 !Undercoordination parameter
 -55.1978 !Triple bond stabilization energy
   0.0000 !Lower Taper-radius
  10.0000 !Upper Taper-radius
   2.8793 !Not used
  33.8667 !Valency undercoordination
   6.0891 !Valency angle/lone pair parameter
   1.0563 !Valency angle
   2.0384 !Valency angle parameter
   6.1431 !Not used
   6.9290 !Double bond/angle parameter
   0.3989 !Double bond/angle parameter: overcoord
   3.9954 !Double bond/angle parameter: overcoord
  -2.4837 !Not used
   5.7796 !Torsion/BO parameter
  10.0000 !Torsion overcoordination
   1.9487 !Torsion overcoordination
  -1.2327 !Conjugation 0 (not used)
   2.1645 !Conjugation
   1.5591 !vdWaals shielding
   0.1000 !Cutoff for bond order (*100)
   1.7602 !Valency angle conjugation parameter
   0.6991 !Overcoordination parameter
  50.0000 !Overcoordination parameter
   1.8512 !Valency/lone pair parameter
   0.5000 !Not used
  20.0000 !Not used
   5.0000 !Molecular energy (not used)
   0.0000 !Molecular energy (not used)
   0.7903 !Valency angle conjugation parameter
 12    ! Nr of atoms; cov.r; valency;a.m;Rvdw;Evdw;gammaEEM;cov.r2;
         alfa;gammavdW;valency;Eunder;Eover;chiEEM;etaEEM;n.u.
         cov r3;Elp;Heat inc.;n.u.;n.u.;n.u.;n.u.
```

ov/un;val1;n.u.;val3,vval4
C 1.3817 4.0000 12.0000 1.8903 0.1838 0.6387 1.1341 4.0000
9.7559 2.1346 4.0000 34.9350 79.5548 4.9218 6.0000 0.0000
1.2114 0.0000 202.2900 88.9539 34.9289 13.5366 0.8563 0.0000
-2.8983 2.5000 1.0560 44.0000 2.9663 0.0000 0.0000 0.0000
H 0.8930 1.0000 1.0080 1.3550 0.0930 0.8203 -0.1000 1.0000
8.2230 33.2894 1.0000 0.0000 121.1250 3.7248 9.6093 1.0000
-0.1000 0.0000 55.1870 83.0408 2.4197 0.0003 1.0698 0.0000
-19.4571 4.2733 1.0330 81.0000 2.8793 0.0000 0.0000 0.0000
O 1.2450 2.0000 15.9990 2.3890 0.1000 1.0898 1.0548 6.0000
9.7300 13.8449 4.0000 37.5000 116.0768 8.5000 8.3122 2.0000
0.9049 0.4056 68.0152 3.5027 0.7640 0.0021 0.9745 0.0000
-3.5500 2.9000 1.0493 4.0000 2.9225 0.0000 0.0000 0.0000
Mn 2.2190 7.0000 54.9380 1.9685 0.3855 0.4633 0.1000 6.0000
11.2058 4.1928 4.0000 0.0000 0.0000 -1.0000 6.1911 0.0000
0.1000 0.0000 152.6300 3.4529 0.0722 3.1767 0.8563 0.0000
-25.0000 3.1072 1.0338 8.0000 3.4590 0.0000 0.0000 0.0000
Li 1.9814 1.0000 6.9410 1.8000 0.2939 0.9387 -0.1000 1.0000
9.0616 1.3258 1.0000 0.0000 0.0000 -3.0000 10.0241 0.0000
-1.0000 0.0000 37.5000 5.4409 6.9107 0.1973 0.8563 0.0000
-2.5068 2.2989 1.0338 1.0000 2.8103 1.3000 0.2000 13.0000
F 1.7938 1.0000 18.9984 1.4139 0.3134 0.7750 -0.1000 7.0000
10.3051 15.1397 1.0000 9.2533 0.2000 9.8105 8.6941 0.0000
-1.0000 3.5571 18.0000 6.9821 4.1799 1.0561 0.0000 0.0000
-6.3417 2.6656 1.0493 4.0000 2.9225 0.0000 0.0000 0.0000
P 1.5994 3.0000 30.9738 1.7000 0.1743 1.0385 1.3000 5.0000
9.1909 14.2932 5.0000 0.0000 0.0000 0.9528 7.9121 0.0000
-1.0000 10.2596 1.5000 0.2205 16.7429 15.9629 0.0000 0.0000
-2.5000 1.6114 1.0338 5.0000 2.8793 0.0000 0.0000 0.0000
Ni -0.1000 2.0000 1.0080 2.0000 0.0000 1.0000 -0.1000 6.0000
10.0000 2.5000 4.0000 0.0000 0.0000 8.5000 1.5000 0.0000
-0.1000 0.0000 -2.3700 8.7410 13.3640 0.6690 0.9745 0.0000
-11.0000 2.7466 1.0338 6.2998 2.8793 0.0000 0.0000 0.0000
Al -0.1000 2.0000 1.0080 2.0000 0.0000 1.0000 -0.1000 6.0000
10.0000 2.5000 4.0000 0.0000 0.0000 8.5000 1.5000 0.0000
-0.1000 0.0000 -2.3700 8.7410 13.3640 0.6690 0.9745 0.0000
-11.0000 2.7466 1.0338 6.2998 2.8793 0.0000 0.0000 0.0000
Cu 1.9202 2.0000 63.5460 1.9221 0.2826 1.0000 0.1000 1.0000
10.9889 100.0000 1.0000 0.0000 0.0000 2.7875 6.0000 0.0000
-1.0000 0.0000 80.7000 34.9555 0.4988 0.0000 0.8563 0.0000
-5.1872 3.1491 1.0000 4.0000 2.5791 0.0000 0.0000 0.0000
N 1.2333 3.0000 14.0000 2.1263 0.1207 1.0000 1.1748 5.0000
9.9865 13.2428 4.0000 26.4087 100.0000 6.3619 6.9188 2.0000
1.0433 3.9512 119.9837 0.7170 7.4321 2.3377 0.9745 0.0000
-3.5800 4.0000 1.0183 4.0000 2.8793 0.0000 0.0000 0.0000
S 1.9673 2.0000 32.0600 2.1729 0.3000 1.0336 1.5359 6.0000
10.3008 4.9055 4.0000 52.9998 112.1416 6.5000 8.2545 2.0000
1.4601 9.7177 71.1843 5.7487 23.2859 12.7147 0.9745 0.0000
-11.0000 2.7466 1.0338 6.2998 2.8793 0.0000 0.0000 0.0000
42 ! Nr of bonds; Edis1;LPpen;n.u.;pbe1;pbo5;13corr;pbo6
pbe2;pbo3;pbo4;Etrip;pbo1;pbo2;ovcorr
1 1 158.2004 99.1897 78.0000 -0.7738 -0.4550 1.0000 37.6117 0.4147

0.4590 -0.1000 9.1628 1.0000 -0.0777 6.7268 1.0000 0.0000
1 2 169.4760 0.0000 0.0000 -0.6083 0.0000 1.0000 6.0000 0.7652
5.2290 1.0000 0.0000 1.0000 -0.0553 6.9316 0.0000 0.0000
2 2 153.3934 0.0000 0.0000 -0.4600 0.0000 1.0000 6.0000 0.7300
6.2500 1.0000 0.0000 1.0000 -0.0790 6.0552 0.0000 0.0000
1 3 115.3161 127.1562 61.7072 0.5141 -0.3474 1.0000 18.9948 0.9954
1.5618 -0.3414 8.9489 1.0000 -0.1628 5.6821 0.0000 0.0000
3 3 142.2858 145.0000 50.8293 0.2506 -0.1000 1.0000 29.7503 0.6051
0.3451 -0.1055 9.0000 1.0000 -0.1225 5.5000 1.0000 0.0000
2 3 160.0000 0.0000 0.0000 -0.5725 0.0000 1.0000 6.0000 0.5626
1.1150 1.0000 0.0000 0.0000 -0.0920 4.2790 0.0000 0.0000
1 4 49.3020 10.0000 0.0000 -1.0302 -0.3000 1.0000 36.0000 -0.0099
1.8910 -0.4204 7.6871 1.0000 -0.0880 6.6676 1.0000 0.0000
2 4 101.9444 0.0000 0.0000 -0.2285 -0.3000 0.0000 36.0000 1.0271
7.0832 -0.1532 19.8594 1.0000 -0.0836 4.7210 0.0000 0.0000
3 4 88.0134 107.3137 0.0000 0.4680 -0.5000 1.0000 50.0000 2.0000
7.5815 -0.4509 7.6953 1.0000 -0.0952 5.1424 1.0000 0.0000
4 4 75.6824 0.0000 0.0000 -0.9934 -0.3000 0.0000 16.0000 0.2050
2.2857 -0.3000 16.0000 1.0000 -0.1230 8.7005 0.0000 0.0000
1 5 54.6610 -0.0200 0.0000 -0.8605 -0.5000 0.0000 35.0000 0.3953
0.6908 -0.2500 11.9965 1.0000 -0.0668 9.0596 0.0000 0.0000
2 5 59.2034 0.0000 0.0000 0.1240 0.0000 0.0000 6.0000 0.4000
1.0000 0.0000 12.0000 1.0000 -0.0565 4.9575 0.0000 0.0000
3 5 223.9629 10.0000 0.0000 -0.8805 0.0000 1.0000 6.0000 0.2178
4.2326 -0.1048 7.0167 1.0000 -0.0867 4.2825 0.0000 0.0000
4 5 100.0000 0.0000 0.0000 -3.1025 0.3000 0.0000 26.0000 2.0000
3.2823 0.0000 12.0000 1.0000 -0.0800 6.9752 0.0000 0.0000
5 5 34.3154 0.0000 0.0000 0.5995 0.3000 0.0000 26.0000 0.5445
0.5752 0.0000 12.0000 1.0000 -0.1382 4.5000 0.0000 0.0000
2 6 233.4343 0.0000 0.0000 -0.4237 -0.3500 1.0000 25.0000 1.9689
4.7870 -0.2500 15.0000 1.0000 -0.1288 4.9159 1.0000 0.0000
3 6 197.9420 0.0000 0.0000 -0.7552 -0.3500 1.0000 25.0000 0.2137
9.5888 -0.2500 15.0000 1.0000 -0.1477 7.8947 1.0000 0.0000
4 6 182.8718 14.3332 0.0000 -0.3452 0.3022 0.0000 6.0000 0.3585
2.6684 -0.1484 11.8165 1.0000 -0.0940 5.8491 0.0000 0.0000
6 6 124.4206 0.0000 0.0000 -1.0188 -0.3500 1.0000 25.0000 0.0863
8.4257 -0.2500 15.0000 1.0000 -0.2337 8.1452 1.0000 0.0000
1 6 230.5926 0.0000 0.0000 -0.9278 0.0000 1.0000 6.0000 1.0927
7.1644 1.0000 0.0000 1.0000 -0.0732 5.9745 0.0000 0.0000
5 6 92.6032 0.0000 0.0000 -0.5454 -0.5582 0.0000 44.9378 0.3238
1.0563 -0.2500 15.0000 1.0000 -0.1206 4.0000 0.0000 0.0000
1 7 110.0000 92.0000 0.0000 0.2171 -0.1418 1.0000 13.1260 0.6000
0.3601 -0.1310 10.7257 1.0000 -0.0869 5.3302 1.0000 0.0000
2 7 0.1466 0.0000 0.0000 0.2250 -0.1418 1.0000 13.1260 0.6000
0.3912 -0.1310 0.0000 1.0000 -0.1029 9.3302 0.0000 0.0000
3 7 202.5868 164.1808 0.0000 0.5506 -0.5000 1.0000 25.0000 0.4300
0.0912 -0.1285 16.0342 1.0000 -0.2008 6.2678 1.0000 0.0000
7 7 0.0000 0.0000 0.0000 0.2171 -0.5000 1.0000 35.0000 0.6000
0.5000 -0.5000 20.0000 1.0000 -0.2000 10.0000 1.0000 0.0000
6 7 191.3390 10.2597 0.0000 0.9085 -0.5000 1.0000 0.0000 0.1616
0.0940 -0.1039 10.0002 1.0000 -0.1073 9.9960 1.0000 0.0000
5 7 0.0000 0.0000 0.0000 0.5000 -0.2000 0.0000 16.0000 0.5000

```
       1.0001  -0.2000  15.0000   1.0000  -0.1000  10.0000   0.0000   0.0000
  4  7   0.0000   0.0000   0.0000   0.5000  -0.2000   0.0000  16.0000   0.5000
       1.0001  -0.2000  15.0000   1.0000  -0.1000  10.0000   0.0000   0.0000
  4 10  73.8502   0.9843  -0.0997  -0.1315  -0.2263   0.1550  14.8700   0.4174
       0.3101  -0.2125  14.9850   1.0420  -0.1266   6.0687  -0.5253   0.4960
  1 10   0.0000   0.0000   0.0000   0.2000  -0.1418   1.0000  13.1260   0.5000
       0.5000  -0.2000  20.0000   1.0000  -0.1000   9.0000   0.0000   0.0000
  2 10  73.6182   0.0000   0.0000  -0.5004  -0.2000   0.0000  16.0000   0.3418
       9.0000  -0.2000  15.0000   1.0000  -0.1015   5.7850   0.0000   0.0000
  3 10  81.4346   0.0000   0.0000  -0.1594  -0.3000   1.0000  36.0000   0.0025
       0.2904  -0.2500  12.0000   1.0000  -0.0742   9.3638   0.0000   0.0000
 11 10  96.5322   0.0000   0.0000   0.9970  -0.3000   1.0000  36.0000   0.5095
       0.7247  -0.2500  12.0000   1.0000  -0.1175   9.9985   0.0000   0.0000
 10 10  73.6263   0.0000   0.0000   0.0209  -0.2000   0.0000  16.0000   0.3414
       0.4703  -0.2000  15.0000   1.0000  -0.1319   5.9254   0.0000   0.0000
  1 11 164.8857 144.1480  94.6331  -1.4899  -0.4639   1.0000  27.3065   0.2354
       0.3170  -0.3518   7.1266   1.0000  -0.2521   4.5741   1.0000   0.0000
  3 11 128.8596 167.8643  40.0000   0.3819  -0.1539   1.0000  34.9972   0.1900
       1.0110  -0.3716   7.0805   1.0000  -0.1265   6.8843   1.0000   0.0000
 11 11 160.1592  82.5526 153.9884   0.4110  -0.0934   1.0000  12.4304   0.5899
       0.1538  -0.1473  11.9187   1.0000  -0.0753   5.4371   1.0000   0.0000
  2 11 211.0748   0.0000   0.0000  -0.3810   0.0000   1.0000   6.0000   0.4878
       2.7875   1.0000   0.0000   1.0000  -0.1608   5.6332   0.0000   0.0000
  1 17 130.0000   0.0000   0.0000   0.2171  -0.1418   1.0000  13.1260   0.6000
       0.3601  -0.1310  10.7257   1.0000  -0.0869   5.3302   1.0000   0.0000
  4 11   0.0000   0.0000   0.0000   0.5000  -0.2000   0.0000  16.0000   0.5000
       1.0001  -0.2000  15.0000   1.0000  -0.1000  10.0000   0.0000   0.0000
  2 12 143.4377   0.0000   0.0000  -0.2944   0.0000   1.0000   6.0000   0.6034
       9.5627   1.0000   0.0000   1.0000  -0.0516   7.0960   1.0000   0.0000
  3 12   0.0000   0.0000   0.0000   0.5563  -0.4038   1.0000  49.5611   0.6000
       0.4259  -0.4577  12.7569   1.0000  -0.1100   7.1145   1.0000   0.0000
 26     ! Nr of off-diagonal terms; Ediss;Ro;gamma;rsigma;rpi;rpi2
  1  2   0.1239   1.4004   9.8467   1.1210  -1.0000  -1.0000
  2  3   0.0283   1.2885  10.9190   0.9215  -1.0000  -1.0000
  1  3   0.0503   1.8006  10.2114   1.3492   1.1992   1.0506
  1  4   0.0475   1.6810   8.8902   1.5153   1.0000  -1.0000
  2  4   0.1017   1.3259  12.5406   1.4534  -1.0000  -1.0000
  3  4   0.1121   2.0879  11.2110   1.5063   1.4795  -1.0000
  1  5   0.0270   2.4124  11.4640   1.7840   1.0000   1.0000
  2  5   0.1149   1.4658  11.0886   1.3337  -1.0000  -1.0000
  3  5   0.0687   1.8765  12.0451   1.2928  -1.0000  -1.0000
  4  5   7.1011   0.9961  40.6852   1.7459  -1.0000  -1.0000
  2  6   0.1087   1.8734   9.1166   1.0000  -1.0000  -1.0000
  3  6   0.1714   1.6576  10.7298   1.5553  -1.0000  -1.0000
  4  6   0.0837   1.9512  12.2762   1.4795  -1.0000  -1.0000
  5  6   0.1092   1.7165  10.1139   1.5274  -1.0000  -1.0000
  1  6   0.1485   1.3609  11.8373   1.3335   1.4000  -1.0000
  6  7   0.1435   1.0021   9.7624   1.7440  -1.0000  -1.0000
  2 11   0.1622   1.3103   9.7014   1.0582  -1.0000  -1.0000
  1 11   0.1925   1.8585   9.7233   1.3420   1.2492   1.1328
  3 11   0.1916   1.8995   9.3215   1.4412   1.0485   1.2276
  1 10   0.0500   1.7500  12.3500   0.1000  -1.0000  -1.0000
```

```
  2 10   0.0300   1.5200  12.5000   0.1000  -1.0000  -1.0000
  3 10   0.0348   1.7637  12.3562   1.7228  -1.0000  -1.0000
  4 10   8.9905   1.0046  40.9082   1.8159  -1.0226  -1.5487
 11 10   0.0478   1.7704  12.8051   1.6100  -1.0000  -1.0000
  2 12   0.0764   1.5838  10.1462   1.4206  -1.0000  -1.0000
  3 12   0.1022   1.9887  10.0605   1.5799   1.4000  -1.0000
114    ! Nr of angles;at1;at2;at3;Thetao,o;ka;kb;pv1;pv2
  1  1  1  59.0573  30.7029   0.7606   0.0000   0.7180   6.2933   1.1244
  1  1  2  65.7758  14.5234   6.2481   0.0000   0.5665   0.0000   1.6255
  2  1  2  70.2607  25.2202   3.7312   0.0000   0.0050   0.0000   2.7500
  1  2  2   0.0000   0.0000   6.0000   0.0000   0.0000   0.0000   1.0400
  1  2  1   0.0000   3.4110   7.7350   0.0000   0.0000   0.0000   1.0400
  2  2  2   0.0000  27.9213   5.8635   0.0000   0.0000   0.0000   1.0400
  1  1  3  54.7427  21.1992   1.0613   0.0000   2.9950  58.6562   1.1232
  3  1  3  78.6632  16.3065   6.3613 -19.9300   1.5183   0.0000   2.2234
  2  1  3  50.0000  12.9103   2.5311   0.0000   0.1000   0.0000   1.0000
  1  3  1  71.6401  45.0000   1.2667   0.0000   2.8294   0.0000   1.0000
  1  3  3  76.3686  44.8665   1.9461   0.0000   1.0572  68.1072   1.8676
  3  3  3  89.9293  15.8855   2.0229   0.0000   2.9881   0.0000   1.0237
  1  3  2  90.0000   6.6459   5.2255   0.0000   1.3111   0.0000   3.0000
  2  3  3  75.6935  50.0000   2.0000   0.0000   1.0000   0.0000   1.1680
  2  3  2  85.8000   9.8453   2.2720   0.0000   2.8635   0.0000   1.5800
  1  2  3   0.0000  16.7302   1.1143   0.0000   0.0000   0.0000   1.0000
  3  2  3   0.0000  15.0000   2.8900   0.0000   0.0000   0.0000   2.8774
  2  2  3   0.0000   8.5744   3.0000   0.0000   0.0000   0.0000   1.0421
  3  4  3  29.9309   3.6478   2.2492   0.0000   1.1980   0.0000   2.3810
  4  3  4   3.3622  10.1592   2.3865   0.0000   3.2489   0.0000   2.6812
  3  3  4  11.7872  50.0000   5.8383   0.0000   4.6977   0.0000   1.0000
  3  4  4  15.3871   4.8803   8.3929   0.0000   4.1095   0.0000   3.4321
  1  3  4  90.0000  11.8185   3.0517   0.0000   2.1225   0.0000   1.0000
  2  5  2  25.9881   0.0100   1.8827   0.0000   0.6581   0.0000   1.1500
  5  2  5   0.0000   3.6249   1.0000   0.0000   1.0000   0.0000   1.2500
  3  5  3  10.0000   0.1000   1.0302   0.0000   1.0000   0.0000   1.0000
  3  3  5 106.0264   7.0152   0.2086   0.0000   0.0100   0.0000   2.5367
  1  3  5  92.0242   0.1000  10.0000   0.0000   2.8844   0.0000   1.1706
  3  1  5 100.2536   0.0100   4.2329   0.0000   1.7872   0.0000   2.3488
  5  3  5  70.3878   3.0984   2.8121   0.0000   0.9139   0.0000   1.9378
  3  2  6   0.0000   1.8088   0.0100   0.0000   0.0000   0.0000   1.2229
  2  6  2   0.0000   5.6384   1.3636   0.0000   0.4652   0.0000   1.0400
  6  4  6  19.0109  16.8964  13.2508   0.0000  -0.3146   0.0000   1.3785
  6  6  4  35.8369  25.7536   3.2681   0.0000   3.0259   0.0000   1.0179
  3  4  6  23.3113  15.3421   1.7515   0.0000   2.0263   0.0000   1.3581
  5  4  3  59.8446  13.2857   0.3263   0.0000   4.0719   0.0000   1.1745
  2  4  3 284.7908  48.6239   1.6168   0.0000   7.3083   0.0000  14.6937
  4  3  2  48.8774  12.4454   1.0604   0.0000   4.1897   0.0000   1.0077
  5  3  2  52.6599  13.3819   1.0695   0.0000   4.3732   0.0000   1.0078
  6  2  6  10.0000   7.7335   1.0000   0.0000   1.2806   0.0000   1.0400
  2  6  6  10.0000  10.0000   3.8265   0.0000   1.0000   0.0000   1.0400
  6  6  6  59.0573  30.7029   0.7606   0.0000   0.7180   6.2933   1.1244
  2  2  6  10.0000   7.2139   3.7598   0.0000   1.0000   0.0000   1.7496
  6  1  6  66.7242  16.7256   8.4462   0.0000   1.0455   0.0000   3.9255
  6  1  2  70.0840  25.3540   3.4508   0.0000   1.0000   0.0000   3.0000
```

1	1	6	65.7758	14.5234	6.2481	0.0000	1.0000	0.0000	1.6255
1	6	6	10.0000	2.3487	6.0000	0.0000	5.0000	0.0000	1.0000
1	6	1	10.0000	3.4110	7.7350	0.0000	1.0000	0.0000	1.0400
1	10	1	29.1655	3.3035	0.2000	0.0000	1.1221	0.0000	1.0562
1	1	10	59.8697	2.8115	1.9262	0.0000	0.7602	0.0000	1.4056
1	10	10	25.4591	15.9430	0.9664	0.0000	2.2242	0.0000	1.1088
10	1	10	88.6279	26.0015	1.0328	0.0000	0.2361	0.0000	2.0576
2	1	10	47.3695	16.9204	4.1052	0.0000	0.1000	0.0000	1.0050
2	10	2	34.1965	6.6782	6.5943	0.0000	1.3895	0.0000	1.5365
2	2	10	0.1000	30.0000	3.4094	0.0000	2.4379	0.0000	1.5166
10	2	10	0.0000	8.2994	5.7832	0.0000	2.9873	0.0000	1.7716
2	10	10	21.2590	6.5954	0.9951	0.0000	2.8006	0.0000	1.0000
2	10	10	180.0000	-6.9970	24.3956	0.0000	0.7878	0.0000	1.3672
1	3	10	90.0000	12.8684	1.4601	0.0000	0.8757	0.0000	1.0000
3	1	10	18.8567	24.3753	3.9647	0.0000	0.1000	0.0000	1.5314
3	10	3	79.7335	0.0100	0.1392	0.0000	0.4968	0.0000	2.1948
10	3	10	57.6787	4.8566	2.5768	0.0000	0.7552	0.0000	1.0000
2	3	10	59.4556	10.2025	0.7481	0.0000	1.4521	0.0000	1.0000
3	3	10	73.6721	32.6330	1.7223	0.0000	1.0221	0.0000	1.4351
3	10	10	65.7545	5.6268	4.0645	0.0000	1.7794	0.0000	2.6730
3	2	10	0.0000	4.6026	2.5343	0.0000	0.7284	0.0000	1.1051
2	10	3	34.0653	20.1868	4.7461	0.0000	0.1000	0.0000	1.6752
10	4	3	61.5209	15.0852	1.3838	1.5003	4.3494	2.0812	1.5588
1	1	11	71.3734	24.6929	0.9924	0.0000	1.1834	0.0000	3.0000
3	1	11	81.5693	44.4068	1.6150	0.0000	1.1019	0.0000	1.0000
1	11	11	90.0000	13.4845	0.1000	0.0000	1.1155	0.0000	1.0178
2	1	11	71.9367	34.0344	1.2105	0.0000	0.2000	0.0000	2.6625
1	2	11	0.0000	0.0019	6.3000	0.0000	0.0000	0.0000	1.0400
1	3	11	69.8607	36.7169	2.2351	0.0000	2.9000	0.0000	2.6086
3	3	11	84.3532	26.7243	1.3018	0.0000	2.9000	0.0000	1.3204
11	3	11	69.9422	27.1979	2.3346	0.0000	3.0072	0.0000	1.0000
2	3	11	72.4215	33.3862	6.7583	0.0000	0.1000	0.0000	1.0251
1	11	1	78.5107	22.7928	1.2062	0.0000	2.8702	0.0000	1.0000
1	11	3	74.7913	29.2724	1.1363	0.0000	2.8701	0.0000	1.5851
1	11	11	71.2090	26.4557	1.5068	0.0000	2.8701	0.0000	2.0738
3	11	3	76.5783	20.2288	2.5796	-18.0069	3.0701	0.0000	1.0000
3	11	11	76.0864	25.6861	1.6009	-0.9193	3.0117	0.0000	1.3343
11	11	11	73.1924	30.7646	1.8832	0.0000	2.9983	0.0000	2.6751
1	11	2	66.6089	10.6194	3.4894	0.0000	0.2025	0.0000	3.0000
2	11	3	77.3833	39.8173	1.5537	0.0000	0.3956	0.0000	3.0000
2	11	11	80.8329	27.3636	1.4942	0.0000	0.5437	0.0000	1.0497
2	11	2	81.3624	12.4645	5.8393	0.0000	0.1000	0.0000	1.0000
1	2	11	0.0000	1.2589	3.0933	0.0000	0.0000	0.0000	1.9707
3	2	11	0.0000	0.0100	0.1000	0.0000	0.0000	0.0000	2.9322
11	2	11	0.0000	0.2836	0.1652	0.0000	0.0000	0.0000	2.0201
2	2	3	0.0000	8.5744	3.0000	0.0000	0.0000	0.0000	1.0421
2	2	11	0.0000	0.0019	6.0000	0.0000	0.0000	0.0000	1.0400
3	10	3	96.2265	4.5610	12.0000	0.0000	0.3211	0.0000	1.5204
3	10	3	0.0000	9.1552	7.9919	0.0000	0.1660	0.0000	1.5386
10	3	10	100.0000	10.1065	6.0000	0.0000	1.0000	0.0000	3.6601
2	3	10	55.0417	3.5032	3.9979	0.0000	1.5171	0.0000	1.0400
3	3	10	70.0000	30.0000	2.0000	0.0000	1.0000	0.0000	1.2500

```
 3 10 10  66.7783  14.3146   0.7911   0.0000   1.0000   0.0000   1.2333
 3 10 11 100.0000  28.1532  12.0000   0.0000   0.2932   0.0000   1.6489
 3 10 11   0.0000  22.7457   2.9039   0.0000   0.5593   0.0000   1.9764
11 10 11  87.0081  27.6432   3.9735   0.0000   4.0000   0.0000   1.4578
11 10 11   0.0000  22.8998   3.1077   0.0000   3.0000   0.0000   1.0696
10 11 10 100.0000  10.1065   6.0000   0.0000   1.0000   0.0000   3.6601
 2 11 10  80.0000   3.5601   3.3645   0.0000   1.5171   0.0000   1.0400
 3 11 10  70.0000  30.0000   2.0000   0.0000   1.0000   0.0000   1.2500
11  3 10  70.0000  30.0000   2.0000   0.0000   1.0000   0.0000   1.2500
11 11 10  70.0000  30.0000   2.0000   0.0000   1.0000   0.0000   1.2500
11 10 10  66.7783  14.3146   0.7911   0.0000   1.0000   0.0000   1.2333
 1  3 10  55.0000  15.0000   1.0000   0.0000   1.0000   0.0000   1.5000
 1 11 10  55.0000  15.0000   1.0000   0.0000   1.0000   0.0000   1.5000
 3 12  3  77.0699  39.4349   2.1313 -30.0000   0.9567   0.0000   1.1483
 2 12  2  85.0000  15.1317   2.0000   0.0000   0.5000   0.0000   2.0000
 3  3 12  83.9753  31.0715   3.5590   0.0000   0.8161   0.0000   1.1776
 2  3 12  76.9521  20.0000   2.0903   0.0000   1.0000   0.0000   1.0400
53    ! Nr of torsions;at1;at2;at3;at4;;V1;V2;V3;V2(BO);vconj;n.u;n
 1  1  1  1  -0.2500  34.7453   0.0288  -6.3507  -1.6000   0.0000   0.0000
 1  1  1  2  -0.2500  29.2131   0.2945  -4.9581  -2.1802   0.0000   0.0000
 2  1  1  2  -0.2500  31.2081   0.4539  -4.8923  -2.2677   0.0000   0.0000
 1  1  1  3  -2.5000  25.4016   1.0000  -4.4850  -1.1000   0.0000   0.0000
 2  1  1  3  -0.9763  59.4161   1.0000  -7.7414  -1.0978   0.0000   0.0000
 3  1  1  3  -2.5000  52.7614  -1.0000  -4.0134  -0.8614   0.0000   0.0000
 1  1  3  1  -1.9125  80.0000  -1.0000  -4.5626  -0.9000   0.0000   0.0000
 1  1  3  2   0.6154   8.3019  -0.4870  -2.9336  -0.9000   0.0000   0.0000
 2  1  3  1  -2.5000  80.0000   0.9658  -4.4935  -0.9000   0.0000   0.0000
 2  1  3  2  -1.0000  31.8695   1.0000  -2.6151  -1.1000   0.0000   0.0000
 1  1  3  3   0.7514  34.1941   0.5669  -5.5360  -2.0544   0.0000   0.0000
 2  1  3  3   2.5000  80.0000   1.0000  -2.6841  -2.8274   0.0000   0.0000
 3  1  3  1   0.2515  79.1495  -0.6263  -4.3647  -3.0437   0.0000   0.0000
 3  1  3  2   1.0000  37.1243   1.0000  -2.5000  -3.0476   0.0000   0.0000
 3  1  3  3  -1.0092  41.0504   0.3915  -6.0913  -2.7174   0.0000   0.0000
 1  3  3  1  -1.6378 -11.8357   0.3815  -3.2104  -2.7536   0.0000   0.0000
 1  3  3  2  -2.5000  -9.2805   0.3063  -5.9187  -2.9498   0.0000   0.0000
 2  3  3  2   0.2732 -21.6925  -1.0000  -2.5000  -0.9921   0.0000   0.0000
 1  3  3  3   2.5000 -17.6041   1.0000  -2.5000  -0.9972   0.0000   0.0000
 2  3  3  3  -2.5000  78.0855  -0.8750  -7.8902  -1.2407   0.0000   0.0000
 3  3  3  3  -2.5000 -25.0000   1.0000  -2.5000  -0.9000   0.0000   0.0000
 0  1  2  0   0.0000   0.0000   0.0000   0.0000   0.0000   0.0000   0.0000
 0  2  2  0   0.0000   0.0000   0.0000   0.0000   0.0000   0.0000   0.0000
 0  2  3  0   0.0000   0.1000   0.0200  -2.5415   0.0000   0.0000   0.0000
 0  1  1  0   0.0000  50.0000   0.3000  -4.0000  -2.0000   0.0000   0.0000
 0  3  3  0   0.5511  25.4150   1.1330  -5.1903  -1.0000   0.0000   0.0000
 0  1  6  0   3.3423  30.3435   0.0365  -2.7171   0.0000   0.0000   0.0000
 0  6  6  0  -0.0500  10.0000   0.1565  -2.2006   0.0000   0.0000   0.0000
 0  2  6  0   0.0000   0.0000   0.0000   0.0000   0.0000   0.0000   0.0000
 1  1  1  6  -0.2500  29.2131   0.2945  -4.9581  -2.1802   0.0000   0.0000
 6  1  1  6  -0.2500  31.2081   0.4539  -4.8923  -2.2677   0.0000   0.0000
 6  6  6  6  -0.2500  34.7453   0.0288  -6.3507  -1.6000   0.0000   0.0000
 1  1 11  2   1.6184  23.0932  -0.0288  -6.5396  -1.9825   0.0000   0.0000
 2  1 11  2  -0.5406  63.3144   0.5040  -7.3419  -2.1051   0.0000   0.0000
```

```
  3  1 11  2   1.0000  28.6154   1.0000  -3.2958  -2.5261   0.0000   0.0000
  3  1  1 11  -0.5652  57.5647   0.8258  -4.8580  -0.9511   0.0000   0.0000
 11  1  1 11   0.6625  64.6500   0.1720  -3.7433  -1.7241   0.0000   0.0000
  1  1 11  1   1.0000  11.8739   0.7164  -4.7719  -1.6589   0.0000   0.0000
  3  1 11  1  -1.0000   1.1660   1.0000  -2.7183  -1.8038   0.0000   0.0000
  2  1  1 11   1.0000  27.4158   0.3693  -3.5264  -1.9000   0.0000   0.0000
 11  1 11  2   1.0000  93.1810   1.0000  -6.8476  -2.0202   0.0000   0.0000
  2  1 11  1  -1.0000  90.3346  -0.2760  -7.9897  -1.5996   0.0000   0.0000
  0  1 11  0   0.2176  40.4126   0.3535  -3.9875  -2.0051   0.0000   0.0000
  0  2 11  0   0.0000   0.1032   0.3000  -5.0965   0.0000   0.0000   0.0000
  0  3 11  0   1.1397  61.3225   0.5139  -3.8507  -2.7831   0.0000   0.0000
  0 11 11  0   0.7265  44.3155   1.0000  -4.4046  -2.0000   0.0000   0.0000
 11  1 11 11  -0.0949   8.7582   0.3310  -7.9430  -2.0000   0.0000   0.0000
  2  3 10  3  -1.5000   6.8333  -0.1978  -1.4683   0.0000   0.0000   0.0000
  2  3 10 11  -0.6181   7.1542  -0.0047  -1.6577   0.0000   0.0000   0.0000
  2 11 10  3  -1.5000   1.7820  -1.0000  -5.4916   0.0000   0.0000   0.0000
  2 11 10 11  -0.1959   2.3626  -1.0000  -3.0702   0.0000   0.0000   0.0000
  2  1 11 10   0.0000  10.0000   0.3000  -6.0000  -1.0000   0.0000   0.0000
  0  2 12  0   0.0000   0.0000   0.0000   0.0000   0.0000   0.0000   0.0000
  4   ! Nr of hydrogen bonds;at1;at2;at3;Rhb;Dehb;vhb1
  3  2  3   0.7000  -3.5800   1.4500  19.5000
  3  2 11   2.0758  -4.5000   1.4500  19.5000
 11  2  3   2.1744  -1.6397   1.4500  19.5000
 11  2 11   1.9250  -2.4190   1.4500  19.5000
```

Fe/Mn/O/C/H Force field:

Refinement of:
Fe: c/h/o/water/Fe Aryanpour et al JPC-A 2010 ReaxFF(full)
Mn: Electrode-electrolyte interface layers in lithium ion batteries using reactive force field based molecular dynamics by Sahithya Reddivari, doctoral thesis, 2016, University of Michigan

Reactive MD-force field: Mn/Fe/O/C/H Gomzi et al 2021.

```
 39       ! Number of general parameters
  50.0000 !Overcoordination parameter
   9.5469 !Overcoordination parameter
   1.6725 !Valency angle conjugation parameter
   1.7224 !Triple bond stabilisation parameter
   6.8702 !Triple bond stabilisation parameter
  60.4850 !C2-correction
   1.0588 !Undercoordination parameter
   4.6000 !Triple bond stabilisation parameter
  12.1176 !Undercoordination parameter
  13.3056 !Undercoordination parameter
 -55.1978 !Triple bond stabilization energy
   0.0000!Lower Taper-radius
  10.0000 !Upper Taper-radius
   2.8793 !Not used
  33.8667 !Valency undercoordination
   6.0891 !Valency angle/lone pair parameter
   1.0563 !Valency angle
   2.0384 !Valency angle parameter
   6.1431 !Not used
   6.9290 !Double bond/angle parameter
   0.3989 !Double bond/angle parameter: overcoord
   3.9954 !Double bond/angle parameter: overcoord
  -2.4837 !Not used
   5.7796 !Torsion/BO parameter
  10.0000 !Torsion overcoordination
   1.9487 !Torsion overcoordination
  -1.2327 !Conjugation 0 (not used)
   2.1645 !Conjugation
   1.5591 !vdWaals shielding
   0.1000 !Cutoff for bond order (*100)
   1.7602 !Valency angle conjugation parameter
   0.6991 !Overcoordination parameter
  50.0000 !Overcoordination parameter
   1.8512 !Valency/lone pair parameter
   0.5000 !Not used
  20.0000 !Not used
   5.0000 !Molecular energy (not used)
   0.0000 !Molecular energy (not used)
   0.7903 !Valency angle conjugation parameter
 10    ! Nr of atoms; cov.r; valency;a.m;Rvdw;Evdw;gammaEEM;cov.r2;
            alfa;gammavdW;valency;Eunder;Eover;chiEEM;etaEEM;n.u.
            cov r3;Elp;Heat inc.;n.u.;n.u.;n.u.;n.u.
            ov/un;val1;n.u.;val3,vval4
```

```
C    1.3817   4.0000  12.0000   1.8903   0.1838   0.6387   1.1341   4.0000
     9.7559   2.1346   4.0000  34.9350  79.5548   4.9218   6.0000   0.0000
     1.2114   0.0000 202.2900  88.9539  34.9289  13.5366   0.8563   0.0000
    -2.8983   2.5000   1.0560  44.0000   2.9663   0.0000   0.0000   0.0000
H    0.8930   1.0000   1.0080   1.3550   0.0930   0.8203  -0.1000   1.0000
     8.2230  33.2894   1.0000   0.0000 121.1250   3.7248   9.6093   1.0000
    -0.1000   0.0000  55.1870  83.0408   2.4197   0.0003   1.0698   0.0000
   -19.4571   4.2733   1.0330  81.0000   2.8793   0.0000   0.0000   0.0000
O    1.2450   2.0000  15.9990   2.3890   0.1000   1.0898   1.0548   6.0000
     9.7300  13.8449   4.0000  37.5000 116.0768   8.5000   8.3122   2.0000
     0.9049   0.4056  68.0152   3.5027   0.7640   0.0021   0.9745   0.0000
    -3.5500   2.9000   1.0493   4.0000   2.9225   0.0000   0.0000   0.0000
Mn   2.2190   7.0000  54.9380   1.9685   0.3855   0.4633   0.1000   6.0000
    11.2058   4.1928   4.0000   0.0000   0.0000  -1.0000   6.1911   0.0000
     0.1000   0.0000 152.6300   3.4529   0.0722   3.1767   0.8563   0.0000
   -25.0000   3.1072   1.0338   8.0000   3.4590   0.0000   0.0000   0.0000
Li   1.9814   1.0000   6.9410   1.8000   0.2939   0.9387  -0.1000   1.0000
     9.0616   1.3258   1.0000   0.0000   0.0000  -3.0000  10.0241   0.0000
    -1.0000   0.0000  37.5000   5.4409   6.9107   0.1973   0.8563   0.0000
    -2.5068   2.2989   1.0338   1.0000   2.8103   1.3000   0.2000  13.0000
F    1.7938   1.0000  18.9984   1.4139   0.3134   0.7750  -0.1000   7.0000
    10.3051  15.1397   1.0000   9.2533   0.2000   9.8105   8.6941   0.0000
    -1.0000   3.5571  18.0000   6.9821   4.1799   1.0561   0.0000   0.0000
    -6.3417   2.6656   1.0493   4.0000   2.9225   0.0000   0.0000   0.0000
P    1.5994   3.0000  30.9738   1.7000   0.1743   1.0385   1.3000   5.0000
     9.1909  14.2932   5.0000   0.0000   0.0000   0.9528   7.9121   0.0000
    -1.0000  10.2596   1.5000   0.2205  16.7429  15.9629   0.0000   0.0000
    -2.5000   1.6114   1.0338   5.0000   2.8793   0.0000   0.0000   0.0000
Ni  -0.1000   2.0000   1.0080   2.0000   0.0000   1.0000  -0.1000   6.0000
    10.0000   2.5000   4.0000   0.0000   0.0000   8.5000   1.5000   0.0000
    -0.1000   0.0000  -2.3700   8.7410  13.3640   0.6690   0.9745   0.0000
   -11.0000   2.7466   1.0338   6.2998   2.8793   0.0000   0.0000   0.0000
Al  -0.1000   2.0000   1.0080   2.0000   0.0000   1.0000  -0.1000   6.0000
    10.0000   2.5000   4.0000   0.0000   0.0000   8.5000   1.5000   0.0000
    -0.1000   0.0000  -2.3700   8.7410  13.3640   0.6690   0.9745   0.0000
   -11.0000   2.7466   1.0338   6.2998   2.8793   0.0000   0.0000   0.0000
Fe   1.9506   3.0000  55.8450   2.0308   0.1274   0.7264  -1.0000   3.0000
    11.0534   2.2637   3.0000   0.0000  18.3725   1.2457   7.3021   0.0000
    -1.2000   0.0000  66.4838  30.0000   1.0000   0.0000   0.8563   0.0000
   -16.2040   2.7917   1.0338   6.0000   2.5791   0.0000   0.0000   0.0000
33      ! Nr of bonds; Edis1;LPpen;n.u.;pbe1;pbo5;13corr;pbo6
                         pbe2;pbo3;pbo4;Etrip;pbo1;pbo2;ovcorr
  1  1 158.2004  99.1897  78.0000  -0.7738  -0.4550   1.0000  37.6117   0.4147
         0.4590  -0.1000   9.1628   1.0000  -0.0777   6.7268   1.0000   0.0000
  1  2 169.4760   0.0000   0.0000  -0.6083   0.0000   1.0000   6.0000   0.7652
         5.2290   1.0000   0.0000   1.0000  -0.0553   6.9316   0.0000   0.0000
  2  2 153.3934   0.0000   0.0000  -0.4600   0.0000   1.0000   6.0000   0.7300
         6.2500   1.0000   0.0000   1.0000  -0.0790   6.0552   0.0000   0.0000
  1  3 115.3161 127.1562  61.7072   0.5141  -0.3474   1.0000  18.9948   0.9954
         1.5618  -0.3414   8.9489   1.0000  -0.1628   5.6821   0.0000   0.0000
  3  3 142.2858 145.0000  50.8293   0.2506  -0.1000   1.0000  29.7503   0.6051
         0.3451  -0.1055   9.0000   1.0000  -0.1225   5.5000   1.0000   0.0000
```

2 3 160.0000 0.0000 0.0000 -0.5725 0.0000 1.0000 6.0000 0.5626
1.1150 1.0000 0.0000 0.0000 -0.0920 4.2790 0.0000 0.0000
1 4 49.3020 10.0000 0.0000 -1.0302 -0.3000 1.0000 36.0000 -0.0099
1.8910 -0.4204 7.6871 1.0000 -0.0880 6.6676 1.0000 0.0000
2 4 101.9444 0.0000 0.0000 -0.2285 -0.3000 0.0000 36.0000 1.0271
7.0832 -0.1532 19.8594 1.0000 -0.0836 4.7210 0.0000 0.0000
3 4 88.0134 107.3137 0.0000 0.4680 -0.5000 1.0000 50.0000 2.0000
7.5815 -0.4509 7.6953 1.0000 -0.0952 5.1424 1.0000 0.0000
4 4 75.6824 0.0000 0.0000 -0.9934 -0.3000 0.0000 16.0000 0.2050
2.2857 -0.3000 16.0000 1.0000 -0.1230 8.7005 0.0000 0.0000
1 5 54.6610 -0.0200 0.0000 -0.8605 -0.5000 0.0000 35.0000 0.3953
0.6908 -0.2500 11.9965 1.0000 -0.0668 9.0596 0.0000 0.0000
2 5 59.2034 0.0000 0.0000 0.1240 0.0000 0.0000 6.0000 0.4000
1.0000 0.0000 12.0000 1.0000 -0.0565 4.9575 0.0000 0.0000
3 5 223.9629 10.0000 0.0000 -0.8805 0.0000 1.0000 6.0000 0.2178
4.2326 -0.1048 7.0167 1.0000 -0.0867 4.2825 0.0000 0.0000
4 5 100.0000 0.0000 0.0000 -3.1025 0.3000 0.0000 26.0000 2.0000
3.2823 0.0000 12.0000 1.0000 -0.0800 6.9752 0.0000 0.0000
5 5 34.3154 0.0000 0.0000 0.5995 0.3000 0.0000 26.0000 0.5445
0.5752 0.0000 12.0000 1.0000 -0.1382 4.5000 0.0000 0.0000
2 6 233.4343 0.0000 0.0000 -0.4237 -0.3500 1.0000 25.0000 1.9689
4.7870 -0.2500 15.0000 1.0000 -0.1288 4.9159 1.0000 0.0000
3 6 197.9420 0.0000 0.0000 -0.7552 -0.3500 1.0000 25.0000 0.2137
9.5888 -0.2500 15.0000 1.0000 -0.1477 7.8947 1.0000 0.0000
4 6 182.8718 14.3332 0.0000 -0.3452 0.3022 0.0000 6.0000 0.3585
2.6684 -0.1484 11.8165 1.0000 -0.0940 5.8491 0.0000 0.0000
6 6 124.4206 0.0000 0.0000 -1.0188 -0.3500 1.0000 25.0000 0.0863
8.4257 -0.2500 15.0000 1.0000 -0.2337 8.1452 1.0000 0.0000
1 6 230.5926 0.0000 0.0000 -0.9278 0.0000 1.0000 6.0000 1.0927
7.1644 1.0000 0.0000 1.0000 -0.0732 5.9745 0.0000 0.0000
5 6 92.6032 0.0000 0.0000 -0.5454 -0.5582 0.0000 44.9378 0.3238
1.0563 -0.2500 15.0000 1.0000 -0.1206 4.0000 0.0000 0.0000
1 7 110.0000 92.0000 0.0000 0.2171 -0.1418 1.0000 13.1260 0.6000
0.3601 -0.1310 10.7257 1.0000 -0.0869 5.3302 1.0000 0.0000
2 7 0.1466 0.0000 0.0000 0.2250 -0.1418 1.0000 13.1260 0.6000
0.3912 -0.1310 0.0000 1.0000 -0.1029 9.3302 0.0000 0.0000
3 7 202.5868 164.1808 0.0000 0.5506 -0.5000 1.0000 25.0000 0.4300
0.0912 -0.1285 16.0342 1.0000 -0.2008 6.2678 1.0000 0.0000
7 7 0.0000 0.0000 0.0000 0.2171 -0.5000 1.0000 35.0000 0.6000
0.5000 -0.5000 20.0000 1.0000 -0.2000 10.0000 1.0000 0.0000
6 7 191.3390 10.2597 0.0000 0.9085 -0.5000 1.0000 0.0000 0.1616
0.0940 -0.1039 10.0002 1.0000 -0.1073 9.9960 1.0000 0.0000
5 7 0.0000 0.0000 0.0000 0.5000 -0.2000 0.0000 16.0000 0.5000
1.0001 -0.2000 15.0000 1.0000 -0.1000 10.0000 0.0000 0.0000
4 7 0.0000 0.0000 0.0000 0.5000 -0.2000 0.0000 16.0000 0.5000
1.0001 -0.2000 15.0000 1.0000 -0.1000 10.0000 0.0000 0.0000
1 10 133.0514 0.0000 0.0000 1.0000 -0.3000 1.0000 36.0000 0.0673
0.2350 -0.3500 15.0000 1.0000 -0.1143 4.5217 1.0000 0.0000
2 10 105.0054 0.0000 0.0000 -0.0717 0.0000 0.0000 6.0000 0.0505
0.1000 1.0000 0.0000 1.0000 -0.1216 4.5062 0.0000 0.0000
3 10 65.7713 0.0000 0.0000 0.1366 -0.3000 1.0000 36.0000 0.0494
0.9495 -0.3500 15.0000 1.0000 -0.0555 7.9897 1.0000 0.0000

```
10 10  38.7471   0.0000   0.0000   0.3595  -0.2000   0.0000  16.0000   0.2749
        1.0000  -0.2000  15.0000   1.0000  -0.0771   6.4477   0.0000   0.0000
 4 10  99.7216   4.0265  -0.1816  -2.7044   0.2311  -0.0461  26.2480   1.8600
        4.1052   0.3126  12.5781   0.9850  -0.0903   7.5452  -0.8575  -0.0020
20     ! Nr of off-diagonal terms; Ediss;Ro;gamma;rsigma;rpi;rpi2
 1  2   0.1239   1.4004   9.8467   1.1210  -1.0000  -1.0000
 2  3   0.0283   1.2885  10.9190   0.9215  -1.0000  -1.0000
 1  3   0.0503   1.8006  10.2114   1.3492   1.1992   1.0506
 1  4   0.0475   1.6810   8.8902   1.5153   1.0000  -1.0000
 2  4   0.1017   1.3259  12.5406   1.4534  -1.0000  -1.0000
 3  4   0.1121   2.0879  11.2110   1.5063   1.4795  -1.0000
 1  5   0.0270   2.4124  11.4640   1.7840   1.0000   1.0000
 2  5   0.1149   1.4658  11.0886   1.3337  -1.0000  -1.0000
 3  5   0.0687   1.8765  12.0451   1.2928  -1.0000  -1.0000
 4  5   7.1011   0.9961  40.6852   1.7459  -1.0000  -1.0000
 2  6   0.1087   1.8734   9.1166   1.0000  -1.0000  -1.0000
 3  6   0.1714   1.6576  10.7298   1.5553  -1.0000  -1.0000
 4  6   0.0837   1.9512  12.2762   1.4795  -1.0000  -1.0000
 5  6   0.1092   1.7165  10.1139   1.5274  -1.0000  -1.0000
 1  6   0.1485   1.3609  11.8373   1.3335   1.4000  -1.0000
 6  7   0.1435   1.0021   9.7624   1.7440  -1.0000  -1.0000
 1 10   0.1358   1.8293  10.0425   1.6096  -1.0000  -1.0000
 2 10   0.0640   1.6974  11.5167   1.3517  -1.0000  -1.0000
 3 10   0.0846   1.4284  10.0808   1.8339  -1.0000  -1.0000
 4 10   8.7279   1.0311  41.0444   1.8975  -0.9850  -1.4967
68    ! Nr of angles;at1;at2;at3;Thetao,o;ka;kb;pv1;pv2
 1  1  1  59.0573  30.7029   0.7606   0.0000   0.7180   6.2933   1.1244
 1  1  2  65.7758  14.5234   6.2481   0.0000   0.5665   0.0000   1.6255
 2  1  2  70.2607  25.2202   3.7312   0.0000   0.0050   0.0000   2.7500
 1  2  2   0.0000   0.0000   6.0000   0.0000   0.0000   0.0000   1.0400
 1  2  1   0.0000   3.4110   7.7350   0.0000   0.0000   0.0000   1.0400
 2  2  2   0.0000  27.9213   5.8635   0.0000   0.0000   0.0000   1.0400
 1  1  3  54.7427  21.1992   1.0613   0.0000   2.9950  58.6562   1.1232
 3  1  3  78.6632  16.3065   6.3613 -19.9300   1.5183   0.0000   2.2234
 2  1  3  50.0000  12.9103   2.5311   0.0000   0.1000   0.0000   1.0000
 1  3  1  71.6401  45.0000   1.2667   0.0000   2.8294   0.0000   1.0000
 1  3  3  76.3686  44.8665   1.9461   0.0000   1.0572  68.1072   1.8676
 3  3  3  89.9293  15.8855   2.0229   0.0000   2.9881   0.0000   1.0237
 1  3  2  90.0000   6.6459   5.2255   0.0000   1.3111   0.0000   3.0000
 2  3  3  75.6935  50.0000   2.0000   0.0000   1.0000   0.0000   1.1680
 2  3  2  85.8000   9.8453   2.2720   0.0000   2.8635   0.0000   1.5800
 1  2  3   0.0000  16.7302   1.1143   0.0000   0.0000   0.0000   1.0000
 3  2  3   0.0000  15.0000   2.8900   0.0000   0.0000   0.0000   2.8774
 2  2  3   0.0000   8.5744   3.0000   0.0000   0.0000   0.0000   1.0421
 3  4  3  29.9309   3.6478   2.2492   0.0000   1.1980   0.0000   2.3810
 4  3  4   3.3622  10.1592   2.3865   0.0000   3.2489   0.0000   2.6812
 3  3  4  11.7872  50.0000   5.8383   0.0000   4.6977   0.0000   1.0000
 3  4  4  15.3871   4.8803   8.3929   0.0000   4.1095   0.0000   3.4321
 1  3  4  90.0000  11.8185   3.0517   0.0000   2.1225   0.0000   1.0000
 2  5  2  25.9881   0.0100   1.8827   0.0000   0.6581   0.0000   1.1500
 5  2  5   0.0000   3.6249   1.0000   0.0000   1.0000   0.0000   1.2500
 3  5  3  10.0000   0.1000   1.0302   0.0000   1.0000   0.0000   1.0000
```

```
  3  3  5 106.0264   7.0152   0.2086   0.0000   0.0100   0.0000   2.5367
  1  3  5  92.0242   0.1000  10.0000   0.0000   2.8844   0.0000   1.1706
  3  1  5 100.2536   0.0100   4.2329   0.0000   1.7872   0.0000   2.3488
  5  3  5  70.3878   3.0984   2.8121   0.0000   0.9139   0.0000   1.9378
  3  2  6   0.0000   1.8088   0.0100   0.0000   0.0000   0.0000   1.2229
  2  6  2   0.0000   5.6384   1.3636   0.0000   0.4652   0.0000   1.0400
  6  4  6  19.0109  16.8964  13.2508   0.0000  -0.3146   0.0000   1.3785
  6  6  4  35.8369  25.7536   3.2681   0.0000   3.0259   0.0000   1.0179
  3  4  6  23.3113  15.3421   1.7515   0.0000   2.0263   0.0000   1.3581
  5  4  3  59.8446  13.2857   0.3263   0.0000   4.0719   0.0000   1.1745
  2  4  3 284.7908  48.6239   1.6168   0.0000   7.3083   0.0000  14.6937
  4  3  2  48.8774  12.4454   1.0604   0.0000   4.1897   0.0000   1.0077
  5  3  2  52.6599  13.3819   1.0695   0.0000   4.3732   0.0000   1.0078
  6  2  6  10.0000   7.7335   1.0000   0.0000   1.2806   0.0000   1.0400
  2  6  6  10.0000  10.0000   3.8265   0.0000   1.0000   0.0000   1.0400
  6  6  6  59.0573  30.7029   0.7606   0.0000   0.7180   6.2933   1.1244
  2  2  6  10.0000   7.2139   3.7598   0.0000   1.0000   0.0000   1.7496
  6  1  6  66.7242  16.7256   8.4462   0.0000   1.0455   0.0000   3.9255
  6  1  2  70.0840  25.3540   3.4508   0.0000   1.0000   0.0000   3.0000
  1  1  6  65.7758  14.5234   6.2481   0.0000   1.0000   0.0000   1.6255
  1  6  6  10.0000   2.3487   6.0000   0.0000   5.0000   0.0000   1.0000
  1  6  1  10.0000   3.4110   7.7350   0.0000   1.0000   0.0000   1.0400
  1 10  1  29.1655   3.3035   0.2000   0.0000   1.1221   0.0000   1.0562
  1  1 10  59.8697   2.8115   1.9262   0.0000   0.7602   0.0000   1.4056
  1 10 10  25.4591  15.9430   0.9664   0.0000   2.2242   0.0000   1.1088
 10  1 10  88.6279  26.0015   1.0328   0.0000   0.2361   0.0000   2.0576
  2  1 10  47.3695  16.9204   4.1052   0.0000   0.1000   0.0000   1.0050
  2 10  2  34.1965   6.6782   6.5943   0.0000   1.3895   0.0000   1.5365
  2  2 10   0.1000  30.0000   3.4094   0.0000   2.4379   0.0000   1.5166
 10  2 10   0.0000   8.2994   5.7832   0.0000   2.9873   0.0000   1.7716
  2 10 10  21.2590   6.5954   0.9951   0.0000   2.8006   0.0000   1.0000
  2 10 10 180.0000  -6.9970  24.3956   0.0000   0.7878   0.0000   1.3672
  1  3 10  90.0000  12.8684   1.4601   0.0000   0.8757   0.0000   1.0000
  3  1 10  18.8567  24.3753   3.9647   0.0000   0.1000   0.0000   1.5314
  3 10  3  79.7335   0.0100   0.1392   0.0000   0.4968   0.0000   2.1948
 10  3 10  57.6787   4.8566   2.5768   0.0000   0.7552   0.0000   1.0000
  2  3 10  59.4556  10.2025   0.7481   0.0000   1.4521   0.0000   1.0000
  3  3 10  73.6721  32.6330   1.7223   0.0000   1.0221   0.0000   1.4351
  3 10 10  65.7545   5.6268   4.0645   0.0000   1.7794   0.0000   2.6730
  3  2 10   0.0000   4.6026   2.5343   0.0000   0.7284   0.0000   1.1051
  2 10  3  34.0653  20.1868   4.7461   0.0000   0.1000   0.0000   1.6752
 10  4  3  61.1946  15.2590   1.3088   1.7430   4.3070   1.7812   1.6031
 32    ! Nr of torsions;at1;at2;at3;at4;;V1;V2;V3;V2(BO);vconj;n.u;n
  1  1  1  1  -0.2500  34.7453   0.0288  -6.3507  -1.6000   0.0000   0.0000
  1  1  1  2  -0.2500  29.2131   0.2945  -4.9581  -2.1802   0.0000   0.0000
  2  1  1  2  -0.2500  31.2081   0.4539  -4.8923  -2.2677   0.0000   0.0000
  1  1  1  3  -2.5000  25.4016   1.0000  -4.4850  -1.1000   0.0000   0.0000
  2  1  1  3  -0.9763  59.4161   1.0000  -7.7414  -1.0978   0.0000   0.0000
  3  1  1  3  -2.5000  52.7614  -1.0000  -4.0134  -0.8614   0.0000   0.0000
  1  1  3  1  -1.9125  80.0000  -1.0000  -4.5626  -0.9000   0.0000   0.0000
  1  1  3  2   0.6154   8.3019  -0.4870  -2.9336  -0.9000   0.0000   0.0000
  2  1  3  1  -2.5000  80.0000   0.9658  -4.4935  -0.9000   0.0000   0.0000
```

2	1	3	2	-1.0000	31.8695	1.0000	-2.6151	-1.1000	0.0000	0.0000
1	1	3	3	0.7514	34.1941	0.5669	-5.5360	-2.0544	0.0000	0.0000
2	1	3	3	2.5000	80.0000	1.0000	-2.6841	-2.8274	0.0000	0.0000
3	1	3	1	0.2515	79.1495	-0.6263	-4.3647	-3.0437	0.0000	0.0000
3	1	3	2	1.0000	37.1243	1.0000	-2.5000	-3.0476	0.0000	0.0000
3	1	3	3	-1.0092	41.0504	0.3915	-6.0913	-2.7174	0.0000	0.0000
1	3	3	1	-1.6378	-11.8357	0.3815	-3.2104	-2.7536	0.0000	0.0000
1	3	3	2	-2.5000	-9.2805	0.3063	-5.9187	-2.9498	0.0000	0.0000
2	3	3	2	0.2732	-21.6925	-1.0000	-2.5000	-0.9921	0.0000	0.0000
1	3	3	3	2.5000	-17.6041	1.0000	-2.5000	-0.9972	0.0000	0.0000
2	3	3	3	-2.5000	78.0855	-0.8750	-7.8902	-1.2407	0.0000	0.0000
3	3	3	3	-2.5000	-25.0000	1.0000	-2.5000	-0.9000	0.0000	0.0000
0	1	2	0	0.0000	0.0000	0.0000	0.0000	0.0000	0.0000	0.0000
0	2	2	0	0.0000	0.0000	0.0000	0.0000	0.0000	0.0000	0.0000
0	2	3	0	0.0000	0.1000	0.0200	-2.5415	0.0000	0.0000	0.0000
0	1	1	0	0.0000	50.0000	0.3000	-4.0000	-2.0000	0.0000	0.0000
0	3	3	0	0.5511	25.4150	1.1330	-5.1903	-1.0000	0.0000	0.0000
0	1	6	0	3.3423	30.3435	0.0365	-2.7171	0.0000	0.0000	0.0000
0	6	6	0	-0.0500	10.0000	0.1565	-2.2006	0.0000	0.0000	0.0000
0	2	6	0	0.0000	0.0000	0.0000	0.0000	0.0000	0.0000	0.0000
1	1	1	6	-0.2500	29.2131	0.2945	-4.9581	-2.1802	0.0000	0.0000
6	1	1	6	-0.2500	31.2081	0.4539	-4.8923	-2.2677	0.0000	0.0000
6	6	6	6	-0.2500	34.7453	0.0288	-6.3507	-1.6000	0.0000	0.0000

1 ! Nr of hydrogen bonds;at1;at2;at3;Rhb;Dehb;vhb1

3	2	3	0.7000	-3.5800	1.4500	19.5000

Ni/Mn/O/C/H Force field

Refinement of:
Ni: Supporting information for the manuscript ‘Development and Validation of ReaxFF Reactive Force Field for Hydrocarbon Chemistry Catalyzed by Nickel.’, authors Jonathan E. Mueller, Adri C. T. van Duin and William A. Goddard III
Mn: Electrode-electrolyte interface layers in lithium ion batteries using reactive force field based molecular dynamics by Sahithya Reddivari, doctoral thesis, 2016, University of Michigan

```
Reactive MD-force field: Mn/Ni/O/C/H Gomzi et al 2021.
 39       ! Number of general parameters
   50.0000 !Overcoordination parameter
    9.5469 !Overcoordination parameter
    1.6725 !Valency angle conjugation parameter
    1.7224 !Triple bond stabilisation parameter
    6.8702 !Triple bond stabilisation parameter
   60.4850 !C2-correction
    1.0588 !Undercoordination parameter
    4.6000 !Triple bond stabilisation parameter
   12.1176 !Undercoordination parameter
   13.3056 !Undercoordination parameter
  -55.1978 !Triple bond stabilization energy
    0.0000 !Lower Taper-radius
   10.0000 !Upper Taper-radius
    2.8793 !Not used
   33.8667 !Valency undercoordination
    6.0891 !Valency angle/lone pair parameter
    1.0563 !Valency angle
    2.0384 !Valency angle parameter
    6.1431 !Not used
    6.9290 !Double bond/angle parameter
    0.3989 !Double bond/angle parameter: overcoord
    3.9954 !Double bond/angle parameter: overcoord
   -2.4837 !Not used
    5.7796 !Torsion/BO parameter
   10.0000 !Torsion overcoordination
    1.9487 !Torsion overcoordination
   -1.2327 !Conjugation 0 (not used)
    2.1645 !Conjugation
    1.5591 !vdWaals shielding
    0.1000 !Cutoff for bond order (*100)
    1.7602 !Valency angle conjugation parameter
    0.6991 !Overcoordination parameter
   50.0000 !Overcoordination parameter
    1.8512 !Valency/lone pair parameter
    0.5000 !Not used
   20.0000 !Not used
    5.0000 !Molecular energy (not used)
    0.0000 !Molecular energy (not used)
    0.7903 !Valency angle conjugation parameter
  9    ! Nr of atoms; cov.r; valency;a.m;Rvdw;Evdw;gammaEEM;cov.r2;
```

```
            alfa;gammavdW;valency;Eunder;Eover;chiEEM;etaEEM;n.u.
            cov r3;Elp;Heat inc.;n.u.;n.u.;n.u.;n.u.
            ov/un;val1;n.u.;val3,vval4
 C    1.3817   4.0000  12.0000   1.8903   0.1838   0.6387   1.1341   4.0000
      9.7559   2.1346   4.0000  34.9350  79.5548   4.9218   6.0000   0.0000
      1.2114   0.0000 202.2900  88.9539  34.9289  13.5366   0.8563   0.0000
     -2.8983   2.5000   1.0560  44.0000   2.9663   0.0000   0.0000   0.0000
 H    0.8930   1.0000   1.0080   1.3550   0.0930   0.8203  -0.1000   1.0000
      8.2230  33.2894   1.0000   0.0000 121.1250   3.7248   9.6093   1.0000
     -0.1000   0.0000  55.1870  83.0408   2.4197   0.0003   1.0698   0.0000
    -19.4571   4.2733   1.0330  81.0000   2.8793   0.0000   0.0000   0.0000
 O    1.2450   2.0000  15.9990   2.3890   0.1000   1.0898   1.0548   6.0000
      9.7300  13.8449   4.0000  37.5000 116.0768   8.5000   8.3122   2.0000
      0.9049   0.4056  68.0152   3.5027   0.7640   0.0021   0.9745   0.0000
     -3.5500   2.9000   1.0493   4.0000   2.9225   0.0000   0.0000   0.0000
 Mn   2.2190   7.0000  54.9380   1.9685   0.3855   0.4633   0.1000   6.0000
     11.2058   4.1928   4.0000   0.0000   0.0000  -1.0000   6.1911   0.0000
      0.1000   0.0000 152.6300   3.4529   0.0722   3.1767   0.8563   0.0000
    -25.0000   3.1072   1.0338   8.0000   3.4590   0.0000   0.0000   0.0000
 Li   1.9814   1.0000   6.9410   1.8000   0.2939   0.9387  -0.1000   1.0000
      9.0616   1.3258   1.0000   0.0000   0.0000  -3.0000  10.0241   0.0000
     -1.0000   0.0000  37.5000   5.4409   6.9107   0.1973   0.8563   0.0000
     -2.5068   2.2989   1.0338   1.0000   2.8103   1.3000   0.2000  13.0000
 F    1.7938   1.0000  18.9984   1.4139   0.3134   0.7750  -0.1000   7.0000
     10.3051  15.1397   1.0000   9.2533   0.2000   9.8105   8.6941   0.0000
     -1.0000   3.5571  18.0000   6.9821   4.1799   1.0561   0.0000   0.0000
     -6.3417   2.6656   1.0493   4.0000   2.9225   0.0000   0.0000   0.0000
 P    1.5994   3.0000  30.9738   1.7000   0.1743   1.0385   1.3000   5.0000
      9.1909  14.2932   5.0000   0.0000   0.0000   0.9528   7.9121   0.0000
     -1.0000  10.2596   1.5000   0.2205  16.7429  15.9629   0.0000   0.0000
     -2.5000   1.6114   1.0338   5.0000   2.8793   0.0000   0.0000   0.0000
 Ni   1.8201   2.0000  58.6900   1.9449   0.1880   0.8218   0.1000   2.0000
     12.1594   3.8387   2.0000   0.0000   0.0000   4.8038   7.3852   0.0000
     -1.0000   0.0000  95.6300  50.6786   0.6762   0.0981   0.8563   0.0000
     -3.7733   3.6035   1.0338   8.0000   2.5791   0.0000   0.0000   0.0000
 Al  -0.1000   2.0000   1.0080   2.0000   0.0000   1.0000  -0.1000   6.0000
     10.0000   2.5000   4.0000   0.0000   0.0000   8.5000   1.5000   0.0000
     -0.1000   0.0000  -2.3700   8.7410  13.3640   0.6690   0.9745   0.0000
    -11.0000   2.7466   1.0338   6.2998   2.8793   0.0000   0.0000   0.0000
 33      ! Nr of bonds; Edis1;LPpen;n.u.;pbe1;pbo5;13corr;pbo6
                         pbe2;pbo3;pbo4;Etrip;pbo1;pbo2;ovcorr
  1  1 158.2004  99.1897  78.0000  -0.7738  -0.4550   1.0000  37.6117   0.4147
         0.4590  -0.1000   9.1628   1.0000  -0.0777   6.7268   1.0000   0.0000
  1  2 169.4760   0.0000   0.0000  -0.6083   0.0000   1.0000   6.0000   0.7652
         5.2290   1.0000   0.0000   1.0000  -0.0553   6.9316   0.0000   0.0000
  2  2 153.3934   0.0000   0.0000  -0.4600   0.0000   1.0000   6.0000   0.7300
         6.2500   1.0000   0.0000   1.0000  -0.0790   6.0552   0.0000   0.0000
  1  3 115.3161 127.1562  61.7072   0.5141  -0.3474   1.0000  18.9948   0.9954
         1.5618  -0.3414   8.9489   1.0000  -0.1628   5.6821   0.0000   0.0000
  3  3 142.2858 145.0000  50.8293   0.2506  -0.1000   1.0000  29.7503   0.6051
         0.3451  -0.1055   9.0000   1.0000  -0.1225   5.5000   1.0000   0.0000
  2  3 160.0000   0.0000   0.0000  -0.5725   0.0000   1.0000   6.0000   0.5626
```

1.1150 1.0000 0.0000 0.0000 -0.0920 4.2790 0.0000 0.0000
1 4 49.3020 10.0000 0.0000 -1.0302 -0.3000 1.0000 36.0000 -0.0099
1.8910 -0.4204 7.6871 1.0000 -0.0880 6.6676 1.0000 0.0000
2 4 101.9444 0.0000 0.0000 -0.2285 -0.3000 0.0000 36.0000 1.0271
7.0832 -0.1532 19.8594 1.0000 -0.0836 4.7210 0.0000 0.0000
3 4 88.0134 107.3137 0.0000 0.4680 -0.5000 1.0000 50.0000 2.0000
7.5815 -0.4509 7.6953 1.0000 -0.0952 5.1424 1.0000 0.0000
4 4 75.6824 0.0000 0.0000 -0.9934 -0.3000 0.0000 16.0000 0.2050
2.2857 -0.3000 16.0000 1.0000 -0.1230 8.7005 0.0000 0.0000
1 5 54.6610 -0.0200 0.0000 -0.8605 -0.5000 0.0000 35.0000 0.3953
0.6908 -0.2500 11.9965 1.0000 -0.0668 9.0596 0.0000 0.0000
2 5 59.2034 0.0000 0.0000 0.1240 0.0000 0.0000 6.0000 0.4000
1.0000 0.0000 12.0000 1.0000 -0.0565 4.9575 0.0000 0.0000
3 5 223.9629 10.0000 0.0000 -0.8805 0.0000 1.0000 6.0000 0.2178
4.2326 -0.1048 7.0167 1.0000 -0.0867 4.2825 0.0000 0.0000
4 5 100.0000 0.0000 0.0000 -3.1025 0.3000 0.0000 26.0000 2.0000
3.2823 0.0000 12.0000 1.0000 -0.0800 6.9752 0.0000 0.0000
5 5 34.3154 0.0000 0.0000 0.5995 0.3000 0.0000 26.0000 0.5445
0.5752 0.0000 12.0000 1.0000 -0.1382 4.5000 0.0000 0.0000
2 6 233.4343 0.0000 0.0000 -0.4237 -0.3500 1.0000 25.0000 1.9689
4.7870 -0.2500 15.0000 1.0000 -0.1288 4.9159 1.0000 0.0000
3 6 197.9420 0.0000 0.0000 -0.7552 -0.3500 1.0000 25.0000 0.2137
9.5888 -0.2500 15.0000 1.0000 -0.1477 7.8947 1.0000 0.0000
4 6 182.8718 14.3332 0.0000 -0.3452 0.3022 0.0000 6.0000 0.3585
2.6684 -0.1484 11.8165 1.0000 -0.0940 5.8491 0.0000 0.0000
6 6 124.4206 0.0000 0.0000 -1.0188 -0.3500 1.0000 25.0000 0.0863
8.4257 -0.2500 15.0000 1.0000 -0.2337 8.1452 1.0000 0.0000
1 6 230.5926 0.0000 0.0000 -0.9278 0.0000 1.0000 6.0000 1.0927
7.1644 1.0000 0.0000 1.0000 -0.0732 5.9745 0.0000 0.0000
5 6 92.6032 0.0000 0.0000 -0.5454 -0.5582 0.0000 44.9378 0.3238
1.0563 -0.2500 15.0000 1.0000 -0.1206 4.0000 0.0000 0.0000
1 7 110.0000 92.0000 0.0000 0.2171 -0.1418 1.0000 13.1260 0.6000
0.3601 -0.1310 10.7257 1.0000 -0.0869 5.3302 1.0000 0.0000
2 7 0.1466 0.0000 0.0000 0.2250 -0.1418 1.0000 13.1260 0.6000
0.3912 -0.1310 0.0000 1.0000 -0.1029 9.3302 0.0000 0.0000
3 7 202.5868 164.1808 0.0000 0.5506 -0.5000 1.0000 25.0000 0.4300
0.0912 -0.1285 16.0342 1.0000 -0.2008 6.2678 1.0000 0.0000
7 7 0.0000 0.0000 0.0000 0.2171 -0.5000 1.0000 35.0000 0.6000
0.5000 -0.5000 20.0000 1.0000 -0.2000 10.0000 1.0000 0.0000
6 7 191.3390 10.2597 0.0000 0.9085 -0.5000 1.0000 0.0000 0.1616
0.0940 -0.1039 10.0002 1.0000 -0.1073 9.9960 1.0000 0.0000
5 7 0.0000 0.0000 0.0000 0.5000 -0.2000 0.0000 16.0000 0.5000
1.0001 -0.2000 15.0000 1.0000 -0.1000 10.0000 0.0000 0.0000
4 7 0.0000 0.0000 0.0000 0.5000 -0.2000 0.0000 16.0000 0.5000
1.0001 -0.2000 15.0000 1.0000 -0.1000 10.0000 0.0000 0.0000
1 8 83.5810 9.0383 0.0000 0.2531 -0.2000 1.0000 16.0000 0.0529
1.4085 -0.1113 13.3900 1.0000 -0.1436 4.5683 1.0000 0.0000
2 8 114.7566 0.0000 0.0000 -0.8939 0.0000 1.0000 6.0000 0.1256
0.1054 1.0000 0.0000 1.0000 -0.1196 5.0815 0.0000 0.0000
3 8 118.6999 0.0000 0.0000 -0.1042 -0.2000 1.0000 16.0000 0.1724
0.8280 -0.2500 15.0000 1.0000 -0.1013 5.6326 1.0000 0.0000
4 8 91.2171 -0.8614 -0.0556 -0.3752 -0.2225 -0.1011 16.4000 0.2961

```
   1.2251  -0.2172  15.3527   1.0268  -0.1354   4.2875   0.0348  -0.0200
 8  8  91.2220   0.0000   0.0000  -0.2538  -0.2000   0.0000  16.0000   0.2688
   1.4651  -0.2000  15.0000   1.0000  -0.1435   4.3908   0.0000   0.0000
20    ! Nr of off-diagonal terms; Ediss;Ro;gamma;rsigma;rpi;rpi2
 1  2   0.1239   1.4004   9.8467   1.1210  -1.0000  -1.0000
 2  3   0.0283   1.2885  10.9190   0.9215  -1.0000  -1.0000
 1  3   0.0503   1.8006  10.2114   1.3492   1.1992   1.0506
 1  4   0.0475   1.6810   8.8902   1.5153   1.0000  -1.0000
 2  4   0.1017   1.3259  12.5406   1.4534  -1.0000  -1.0000
 3  4   0.1121   2.0879  11.2110   1.5063   1.4795  -1.0000
 1  5   0.0270   2.4124  11.4640   1.7840   1.0000   1.0000
 2  5   0.1149   1.4658  11.0886   1.3337  -1.0000  -1.0000
 3  5   0.0687   1.8765  12.0451   1.2928  -1.0000  -1.0000
 4  5   7.1011   0.9961  40.6852   1.7459  -1.0000  -1.0000
 2  6   0.1087   1.8734   9.1166   1.0000  -1.0000  -1.0000
 3  6   0.1714   1.6576  10.7298   1.5553  -1.0000  -1.0000
 4  6   0.0837   1.9512  12.2762   1.4795  -1.0000  -1.0000
 5  6   0.1092   1.7165  10.1139   1.5274  -1.0000  -1.0000
 1  6   0.1485   1.3609  11.8373   1.3335   1.4000  -1.0000
 6  7   0.1435   1.0021   9.7624   1.7440  -1.0000  -1.0000
 1  8   0.0800   1.7085  10.0895   1.5504   1.4005  -1.0000
 2  8   0.0366   1.7306  11.1019   1.2270  -1.0000  -1.0000
 3  8   0.0504   1.7959  11.7893   1.4423  -1.0000  -1.0000
 4  8   8.6568   1.1322  40.5350   2.0000  -0.9850  -1.5967
70    ! Nr of angles;at1;at2;at3;Thetao,o;ka;kb;pv1;pv2
 1  1  1  59.0573  30.7029   0.7606   0.0000   0.7180   6.2933   1.1244
 1  1  2  65.7758  14.5234   6.2481   0.0000   0.5665   0.0000   1.6255
 2  1  2  70.2607  25.2202   3.7312   0.0000   0.0050   0.0000   2.7500
 1  2  2   0.0000   0.0000   6.0000   0.0000   0.0000   0.0000   1.0400
 1  2  1   0.0000   3.4110   7.7350   0.0000   0.0000   0.0000   1.0400
 2  2  2   0.0000  27.9213   5.8635   0.0000   0.0000   0.0000   1.0400
 1  1  3  54.7427  21.1992   1.0613   0.0000   2.9950  58.6562   1.1232
 3  1  3  78.6632  16.3065   6.3613 -19.9300   1.5183   0.0000   2.2234
 2  1  3  50.0000  12.9103   2.5311   0.0000   0.1000   0.0000   1.0000
 1  3  1  71.6401  45.0000   1.2667   0.0000   2.8294   0.0000   1.0000
 1  3  3  76.3686  44.8665   1.9461   0.0000   1.0572  68.1072   1.8676
 3  3  3  89.9293  15.8855   2.0229   0.0000   2.9881   0.0000   1.0237
 1  3  2  90.0000   6.6459   5.2255   0.0000   1.3111   0.0000   3.0000
 2  3  3  75.6935  50.0000   2.0000   0.0000   1.0000   0.0000   1.1680
 2  3  2  85.8000   9.8453   2.2720   0.0000   2.8635   0.0000   1.5800
 1  2  3   0.0000  16.7302   1.1143   0.0000   0.0000   0.0000   1.0000
 3  2  3   0.0000  15.0000   2.8900   0.0000   0.0000   0.0000   2.8774
 2  2  3   0.0000   8.5744   3.0000   0.0000   0.0000   0.0000   1.0421
 3  4  3  29.9309   3.6478   2.2492   0.0000   1.1980   0.0000   2.3810
 4  3  4   3.3622  10.1592   2.3865   0.0000   3.2489   0.0000   2.6812
 3  3  4  11.7872  50.0000   5.8383   0.0000   4.6977   0.0000   1.0000
 3  4  4  15.3871   4.8803   8.3929   0.0000   4.1095   0.0000   3.4321
 1  3  4  90.0000  11.8185   3.0517   0.0000   2.1225   0.0000   1.0000
 2  5  2  25.9881   0.0100   1.8827   0.0000   0.6581   0.0000   1.1500
 5  2  5   0.0000   3.6249   1.0000   0.0000   1.0000   0.0000   1.2500
 3  5  3  10.0000   0.1000   1.0302   0.0000   1.0000   0.0000   1.0000
 3  3  5 106.0264   7.0152   0.2086   0.0000   0.0100   0.0000   2.5367
```

```
  1  3  5  92.0242   0.1000  10.0000   0.0000   2.8844   0.0000   1.1706
  3  1  5 100.2536   0.0100   4.2329   0.0000   1.7872   0.0000   2.3488
  5  3  5  70.3878   3.0984   2.8121   0.0000   0.9139   0.0000   1.9378
  3  2  6   0.0000   1.8088   0.0100   0.0000   0.0000   0.0000   1.2229
  2  6  2   0.0000   5.6384   1.3636   0.0000   0.4652   0.0000   1.0400
  6  4  6  19.0109  16.8964  13.2508   0.0000  -0.3146   0.0000   1.3785
  6  6  4  35.8369  25.7536   3.2681   0.0000   3.0259   0.0000   1.0179
  3  4  6  23.3113  15.3421   1.7515   0.0000   2.0263   0.0000   1.3581
  5  4  3  59.8446  13.2857   0.3263   0.0000   4.0719   0.0000   1.1745
  2  4  3 284.7908  48.6239   1.6168   0.0000   7.3083   0.0000  14.6937
  4  3  2  48.8774  12.4454   1.0604   0.0000   4.1897   0.0000   1.0077
  5  3  2  52.6599  13.3819   1.0695   0.0000   4.3732   0.0000   1.0078
  6  2  6  10.0000   7.7335   1.0000   0.0000   1.2806   0.0000   1.0400
  2  6  6  10.0000  10.0000   3.8265   0.0000   1.0000   0.0000   1.0400
  6  6  6  59.0573  30.7029   0.7606   0.0000   0.7180   6.2933   1.1244
  2  2  6  10.0000   7.2139   3.7598   0.0000   1.0000   0.0000   1.7496
  6  1  6  66.7242  16.7256   8.4462   0.0000   1.0455   0.0000   3.9255
  6  1  2  70.0840  25.3540   3.4508   0.0000   1.0000   0.0000   3.0000
  1  1  6  65.7758  14.5234   6.2481   0.0000   1.0000   0.0000   1.6255
  1  6  6  10.0000   2.3487   6.0000   0.0000   5.0000   0.0000   1.0000
  1  6  1  10.0000   3.4110   7.7350   0.0000   1.0000   0.0000   1.0400
  1  8  1  62.5000  16.6806   0.7981   0.0000   0.9630   0.0000   1.0711
  1  1  8  87.6241  12.6504   1.8145   0.0000   0.6154   0.0000   1.5298
  8  1  8 100.0000  40.4895   1.6455   0.0000   0.0100   0.0000   1.7667
  1  8  8   5.0994   3.1824   0.7016   0.0000   0.7465   0.0000   2.2665
  3  8  3  49.6374  27.4219   1.6709   0.0000   0.0369   0.0000   2.5673
  3  3  8  90.0000  31.1442   5.0000   0.0000   0.5686   0.0000   2.0931
  8  3  8  42.4507   5.2354   0.5469   0.0000   1.5478   0.0000   1.0400
  3  8  8  39.2561   2.4303   3.7193   0.0000   0.8924   0.0000   1.9950
  2  8  2 106.3969  30.0000   0.9614   0.0000   1.9664   0.0000   2.2693
  2  2  8   0.0000  26.3327   4.6867   0.0000   0.8177   0.0000   1.0404
  8  2  8   0.0000  60.0000   1.8471   0.0000   0.6331   0.0000   1.8931
  2  8  8  30.3748   1.0000   4.8528   0.0000   0.1019   0.0000   3.1660
  2  8  8 180.0000 -27.2489   8.3752   0.0000   0.8112   0.0000   1.0004
  1  8  2  97.5742  10.9373   2.5200   0.0000   1.8558   0.0000   1.0000
  1  2  8   0.0000   0.2811   1.1741   0.0000   0.9136   0.0000   3.8138
  2  1  8  84.0006  45.0000   0.6271   0.0000   3.0000   0.0000   1.0000
  2  3  8  29.3808  16.2484   2.5832   0.5000   0.0100   0.0000   1.9017
  1  8  3  70.0000  25.0000   1.0000   0.0000   1.0000   0.0000   1.2500
  1  3  8  70.0000  25.0000   1.0000   0.0000   1.0000   0.0000   1.2500
  3  1  8  70.0000  25.0000   1.0000   0.0000   1.0000   0.0000   1.2500
  3  2  8   0.0000   7.1233   1.9895   0.5000   0.3233   0.0000   1.1000
  8  4  3  60.6024  15.0786   1.8619   1.1865   4.6857   1.8189   1.9937
 39    ! Nr of torsions;at1;at2;at3;at4;;V1;V2;V3;V2(BO);vconj;n.u;n
  1  1  1  1  -0.2500  34.7453   0.0288  -6.3507  -1.6000   0.0000   0.0000
  1  1  1  2  -0.2500  29.2131   0.2945  -4.9581  -2.1802   0.0000   0.0000
  2  1  1  2  -0.2500  31.2081   0.4539  -4.8923  -2.2677   0.0000   0.0000
  1  1  1  3  -2.5000  25.4016   1.0000  -4.4850  -1.1000   0.0000   0.0000
  2  1  1  3  -0.9763  59.4161   1.0000  -7.7414  -1.0978   0.0000   0.0000
  3  1  1  3  -2.5000  52.7614  -1.0000  -4.0134  -0.8614   0.0000   0.0000
  1  1  3  1  -1.9125  80.0000  -1.0000  -4.5626  -0.9000   0.0000   0.0000
  1  1  3  2   0.6154   8.3019  -0.4870  -2.9336  -0.9000   0.0000   0.0000
```

2 1 3 1 -2.5000 80.0000 0.9658 -4.4935 -0.9000 0.0000 0.0000
2 1 3 2 -1.0000 31.8695 1.0000 -2.6151 -1.1000 0.0000 0.0000
1 1 3 3 0.7514 34.1941 0.5669 -5.5360 -2.0544 0.0000 0.0000
2 1 3 3 2.5000 80.0000 1.0000 -2.6841 -2.8274 0.0000 0.0000
3 1 3 1 0.2515 79.1495 -0.6263 -4.3647 -3.0437 0.0000 0.0000
3 1 3 2 1.0000 37.1243 1.0000 -2.5000 -3.0476 0.0000 0.0000
3 1 3 3 -1.0092 41.0504 0.3915 -6.0913 -2.7174 0.0000 0.0000
1 3 3 1 -1.6378 -11.8357 0.3815 -3.2104 -2.7536 0.0000 0.0000
1 3 3 2 -2.5000 -9.2805 0.3063 -5.9187 -2.9498 0.0000 0.0000
2 3 3 2 0.2732 -21.6925 -1.0000 -2.5000 -0.9921 0.0000 0.0000
1 3 3 3 2.5000 -17.6041 1.0000 -2.5000 -0.9972 0.0000 0.0000
2 3 3 3 -2.5000 78.0855 -0.8750 -7.8902 -1.2407 0.0000 0.0000
3 3 3 3 -2.5000 -25.0000 1.0000 -2.5000 -0.9000 0.0000 0.0000
0 1 2 0 0.0000 0.0000 0.0000 0.0000 0.0000 0.0000 0.0000
0 2 2 0 0.0000 0.0000 0.0000 0.0000 0.0000 0.0000 0.0000
0 2 3 0 0.0000 0.1000 0.0200 -2.5415 0.0000 0.0000 0.0000
0 1 1 0 0.0000 50.0000 0.3000 -4.0000 -2.0000 0.0000 0.0000
0 3 3 0 0.5511 25.4150 1.1330 -5.1903 -1.0000 0.0000 0.0000
0 1 6 0 3.3423 30.3435 0.0365 -2.7171 0.0000 0.0000 0.0000
0 6 6 0 -0.0500 10.0000 0.1565 -2.2006 0.0000 0.0000 0.0000
0 2 6 0 0.0000 0.0000 0.0000 0.0000 0.0000 0.0000 0.0000
1 1 1 6 -0.2500 29.2131 0.2945 -4.9581 -2.1802 0.0000 0.0000
6 1 1 6 -0.2500 31.2081 0.4539 -4.8923 -2.2677 0.0000 0.0000
6 6 6 6 -0.2500 34.7453 0.0288 -6.3507 -1.6000 0.0000 0.0000
1 1 1 8 0.0000 5.0000 0.4000 -6.0000 0.0000 0.0000 0.0000
8 1 1 8 0.0000 44.3024 0.4000 -4.0000 0.0000 0.0000 0.0000
2 1 1 8 0.0000 21.7038 0.0100 -4.0000 0.0000 0.0000 0.0000
2 1 8 1 0.0000 5.2500 0.0100 -6.0000 0.0000 0.0000 0.0000
1 1 8 1 0.0000 5.1676 0.0100 -5.9539 0.0000 0.0000 0.0000
1 1 8 2 0.0000 5.1676 0.0100 -5.9539 0.0000 0.0000 0.0000
8 3 3 8 0.0509 30.0000 0.5000 -4.0000 0.0000 0.0000 0.0000
4 ! Nr of hydrogen bonds;at1;at2;at3;Rhb;Dehb;vhb1
3 2 3 2.1845 -2.3549 3.0582 19.1627
3 2 4 1.6658 -3.8907 3.0582 19.1627
4 2 3 1.8738 -3.5421 3.0582 19.1627
4 2 4 1.8075 -4.1846 3.0582 19.1627
